# Quantum coherent dynamics in allophycocyanin trimer complex

**DOI:** 10.1126/sciadv.aef1022

**Published:** 2026-09-16

**Authors:** Tianrui Chen, Junhua Zhou, Dehao Yuan, Enhu He, Xiangmei Duan, Fulu Zheng, R. J. Dwayne Miller, Ajay Jha, Hong-Guang Duan

**Affiliations:** ^1^ Department of Physics, School of Physical Science and Technology, Ningbo University, Ningbo 315211, P.R. China.; ^2^ Zhejiang Key Laboratory of Advanced Optical Functional Materials and Devices, Ningbo University, Ningbo 315211, P.R. China.; ^3^ The Departments of Chemistry and Physics, University of Toronto, 80 St. George Street, Toronto M5S 3H6, Canada.; ^4^ Rosalind Franklin Institute, Harwell, Oxfordshire OX11 0QX, UK.; ^5^ Department of Pharmacology, University of Oxford, Oxford OX1 3QT, UK.

## Abstract

For years, scientists have debated whether plants and algae use quantum coherence to enhance the transfer of light energy during photosynthesis. Some studies have suggested that wavelike quantum effects might help energy flow more efficiently between pigments. To investigate this idea, we studied allophycocyanin (APC), a light-harvesting protein complex found in cyanobacteria. Using a combination of ultrafast laser spectroscopic methods and rigorous hierarchical equations-of-motion simulations, we examined both the trimer protein complex and its monomer subunit. The experiments detected rapid oscillations in the optical signals that resembled quantum beats. However, temperature-dependent studies and further analysis showed that these signals were caused by vibrations rather than true quantum superpositions. The excitonic transitions of APC exhibit ultrafast dephasing, within ∼40 femtoseconds at low temperatures and ∼20 femtoseconds at room temperature. This is much shorter than the time required for energy to move between chromophores, which is around 344 femtoseconds. In contrast, certain vibrational motions lasted much longer and remained largely unaffected by temperature. Similar vibrations were observed in both the full protein complex and its isolated subunits, confirming that long-lived oscillations do not rely on interactions between pigments. These findings suggest that long-lived oscillations in APC are predominantly vibrational rather than functionally significant electronic coherence.

## INTRODUCTION

The possibility that quantum effects may contribute to biological function has captivated physicists, chemists, and biologists alike for over a century (*1*, *2*). Among the most studied examples is photosynthesis, the biological process that captures solar energy with near-unity efficiency under ambient conditions (*3*–*5*). This remarkable performance has prompted the long-standing hypothesis that quantum coherence might play a functional role in light harvesting (*6*, *7*). Yet, whether coherent quantum dynamics are preserved and exploited in complex biological environments remains a subject of intense debate (*8*). The term functional coherence refers to a coherence that measurably influences an observable associated with excitation-energy transport, such as the transfer rate, or quantum yield. Consequently, a coherence can only be regarded as functionally relevant if its lifetime is comparable to, or longer than, the timescale of the corresponding population-transfer process.

Photosynthetic light-harvesting complexes operate in noisy, fluctuating environments where interactions with the surrounding protein scaffold and solvent would typically destroy quantum coherence on ultrafast timescales (*9*–*11*). In such “open quantum systems,” the coherence lifetimes are generally too short to affect energy transfer, which occurs over several hundred femtoseconds to picoseconds (*12*, *13*). However, experimental advances over the past two decades, particularly the development of 2DES (*14*–*16*), have revealed signatures of persistent coherence in several pigment-protein complexes, challenging the understanding of rapid decoherence in warm, aqueous environments (*17*, *18*). These discoveries have raised a fundamental question: How, and under what conditions, can quantum coherence survive long enough to influence energy transport in biological systems? A range of mechanisms have been put forward to explain the observed oscillatory features in spectroscopic signals: vibronic mixing between electronic and nuclear degrees of freedom (*19*), non-Markovian environmental effects (*20*), correlated fluctuations within pigment-protein environments (*21*), and transient quantum delocalization across chromophore networks (*22*). These frameworks have inspired new theoretical models and sparked broader interest in coherence-enabled energy transfer (*23*–*26*). Yet despite the conceptual richness of these proposals, a central issue remains unresolved: The precise physical nature of the coherences observed in multidimensional spectroscopy experiments is still under debate (*8*). While oscillatory cross-peak dynamics in 2DES data are frequently interpreted as evidence for long-lived electronic or vibronic superpositions, such assignments are often made in the absence of definitive criteria. The spectral signatures of different types of coherences, i.e., electronic, vibrational, or mixed vibronic, can be remarkably similar, especially under conditions where inhomogeneous broadening, overlapping transitions, and population relaxation all coexist on comparable timescales. As a result, coherent signals detected in 2DES do not unambiguously identify the underlying quantum states involved.

Over the past decade, growing scrutiny has led to a more cautious interpretation of these signals. Several studies have demonstrated that what initially appeared to be persistent electronic coherences could, in fact, be reinterpreted as ground-state vibrational coherences or rephasing artifacts (*8*, *27*–*31*). Moreover, theoretical work has shown that vibronic coherence, which is a superposition of electronic and nuclear motion may explain many observed beatings but only under very specific system-bath coupling (*19*). Even in well-controlled systems such as synthetic dimers or reconstituted protein complexes, observing pure electronic coherence from vibronic or vibrational contributions remains experimentally challenging (*32*). This ambiguity is compounded in biological complexes, where disorder, conformational heterogeneity, and dynamic environmental coupling further complicate the spectral landscape. Despite claims of coherent energy transport in light-harvesting complexes, the field increasingly recognizes that long-lived oscillations are primarily vibrational coherences alone, and the presence of these coherences cannot be taken as proof of their functional nature (*33*, *34*). Instead, a much more stringent combination of time resolution, spectral selectivity, and theoretical modeling is required to rigorously assign coherence origin and assess its role in energy transfer. In this work, we adopt explicit criteria to distinguish vibrational, vibronic, and electronic coherence. Vibrational coherence refers to nuclear wave packets evolving on a single electronic surface, while vibronic coherence involves mixed electronic-vibrational superpositions. Experimentally, these contributions can produce similar oscillatory signatures; therefore, their identification requires combined analysis of temperature dependence, coherence lifetimes relative to electronic dephasing, and comparison with theoretical models. Significant methodological advances in 2DES, particularly through polarization control and separation of rephasing and nonrephasing signal pathways, have further improved the discrimination between electronic, vibrational, and vibronic coherences and now provide important benchmarks for identifying the nature of quantum superpositions in complex systems (*29*, *35*). Related methodological analyses have further shown that the interpretation of beating signals and coherence maps can depend sensitively on pathway contributions and on the distinction between electronic and nuclear coherences, underscoring the need for caution when assigning the origin of oscillatory features in multidimensional spectra (*36*, *37*). In light of this, it is now broadly acknowledged that the initial enthusiasm for coherent dynamics as a dominant mechanism in biological light harvesting may have overstated the evidence. The current frontier lies not in confirming the mere presence of coherence, which is almost inevitable in any coupled quantum system, but in disentangling its character, quantifying its resilience under physiological conditions, and identifying the structural or dynamical features that govern its lifetimes and consequences. To achieve this, it is essential to study systems that strike a balance between complexity and tractability, ones that are biologically relevant but also experimentally and computationally accessible.

Allophycocyanin (APC) trimers present an exemplary platform for probing the interplay between electronic, vibrational, and vibronic coherence. Their well-defined trimeric configuration, intermediate excitonic interactions, and pronounced pigment-specific vibrational features make them highly suited for delineating the boundaries among these coherence regimes. Structural analyses have shown that the APC trimer exhibits a threefold rotational symmetry, with each monomer containing α and β subunits whose Cys84 residues (α84 and β84) are covalently linked to phycocyanobilin (PCB) chromophores, separated by ∼50 Å (*38*). Upon trimer formation, spatial reorganization reduces the interpigment distance between PCBs on adjacent monomers to roughly 20 Å, producing three equivalent α84−β84 pigment pairs. Functionally, APC operates as a core component of the light-harvesting antenna in both blue-green and red algae, efficiently transferring excitation energy from the phycobilisome (*39*) rods to the reaction center (*40*) with an overall quantum efficiency exceeding 90% (*41*). Notably, APC occupies a distinct excitonic parameter regime, in which the excitonic energy gap is on the order of 800 cm^−1^ and approaches characteristic intramolecular vibrational frequencies (see fig. S23 in the Supplementary Materials). This makes APC a stringent system for testing whether near-resonant vibronic conditions can stabilize electronic coherence to an extent that could become functionally relevant in photosynthetic energy transfer. Prior ultrafast spectroscopic and theoretical studies on APC and related phycobiliproteins have already revealed subpicosecond energy-transfer dynamics, anisotropic vibrational signatures, and possible vibronic contributions to exciton delocalization and transport, particularly through comparisons between APC and the closely related C-phycocyanin system (*42*–*46*). Even in this system, recent claims regarding the nature of coherence, for example, those attributing long-lived signals to quantum phase synchronization of vibrational modes, demand careful reevaluation (*47*). Specifically, experimentally observed coherences were assigned as exciton-vibrational coherences in APC trimers, which persist longer than in isolated pigments, attributed to phase synchronization of resonant symmetric vibrational modes within the dimer, which dissipate more slowly than rapidly dephasing antisymmetric modes. While this has been interpreted as structural protection of coherence, prior work indicates conformationally dependent spectral shifts in coupled chromophores with varying conjugation lengths (*48*, *49*). The challenge is not to reinforce a specific mechanistic narrative but to build a robust, temperature-resolved, and theory-supported understanding of coherence in a controlled biological scaffold. In this work, we critically examine the nature and origin of quantum coherence in APC trimers by combining high-resolution 2DES measurements across a wide temperature range (10, 80, and 300 K) with advanced quantum dynamical simulations. By tracking the temperature dependence of coherent beatings, we resolve the timescales and energetic origins of distinct coherence modes. Our work provides an independent and complementary framework for assigning the origin of the observed coherent oscillations through the combination of temperature-dependent spectroscopy and microscopic quantum-dynamical modeling. To interpret these results, we construct a vibronic Hamiltonian explicitly incorporating electronic coupling, intramolecular vibrational modes, and their interactions with a structured thermal bath. Using nonperturbative simulations based on hierarchical equations of motion (HEOM), we reproduce the spectroscopic signatures observed experimentally and provide microscopic insight into the coherent dynamics. Our model captures not only the lifetimes and amplitudes of coherence beatings but also their sensitivity to pigment site energies, coupling strengths, and vibrational resonance conditions.

## RESULTS

**Fig. 1.**
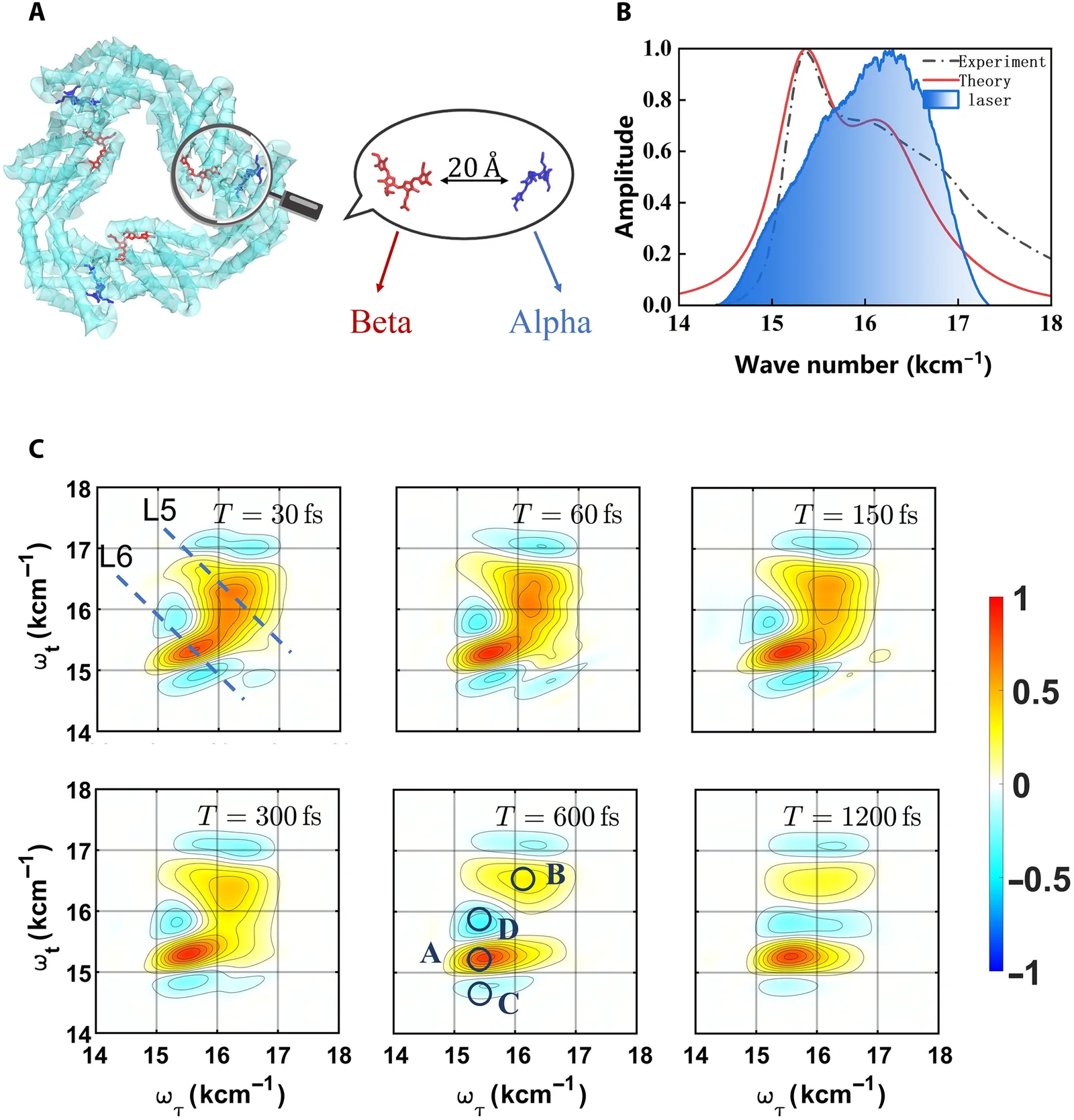
Structure, absorption spectrum, and room-temperature 2DES of APC complex. (**A**) Structure of the APC trimer, showing three α (blue) and β (red) subunit pairs. (**B**) Room-temperature (300 K) absorption spectra: experimental (black dashed) and theoretical (red solid) spectra, with the excitation laser spectrum (blue) overlaid. (**C**) Real part two-dimensional (2D) electronic spectra of APC at 300 K, shown at selected waiting times (*T*, population delays). Two diagonal peaks (A and B) correspond to the α and β excitons, and off-diagonal features (cross peaks C and D) represent excited state absorption features that grow in as energy transfer proceeds. Dashed-dotted lines labeled L5 and L6 in the *T* = 30-fs panel indicate the antidiagonal line cuts used to estimate the homogeneous optical dephasing times of the excitonic transitions.

The proposed excitonic splitting in APC trimer (Fig. 1A) is evident in the linear absorption spectrum (black dashed curve in Fig. 1B), which shows two distinct bands corresponding to the lower-energy (α) and higher-energy (β) excitons. Our calculated spectrum reproduces this absorption profile (red curve), in excellent agreement with experiment. The broad laser pulse spectrum used in our experiments (blue shaded area) spans both absorption peaks, ensuring simultaneous excitation of the two exciton states.

### Two-dimensional electronic spectra

At 300 K, the 2DES resolve the APC excitonic states (Fig. 1C). At waiting time *T* = 30 fs, two intense diagonal peaks appear at (ω_τ_, ω*_t_*) = (15.5, 15.3) kcm^−1^ and (16.25, 16.25) kcm^−1^, matching the absorption band positions. These peaks correspond to the lower (A) and higher (B) exciton transitions and are excited simultaneously by the broadband pulse. By fitting the antidiagonal width of each peak (along line cuts L5 and L6 in Fig. 1C), we estimate homogeneous optical dephasing times of ∼22 and ∼21 fs for the two excitonic transitions (details are provided in the Supplementary Materials, section S1 and fig. S1A). These values provide an estimate of the homogeneous optical dephasing of the excitonic transitions. We note that these linewidths are extracted at a finite waiting time (30 fs), chosen to avoid pulse-overlap artifacts, and in a spectral region where excited-state absorption (ESA) contributions may overlap. Therefore, the extracted values should be regarded as approximate indicators of optical dephasing rather than exact linewidth-lifetime correspondences. At low temperature, however, this linewidth-based estimate should be interpreted with caution, since longer bath memory and non-Markovian effects can make the simple linewidth-lifetime relation only approximate. They nevertheless show that the optical coherences associated with the APC excitonic transitions are lost within only a few optical cycles owing to rapid environmental dephasing. As the population time *T* evolves, clear spectral changes signal downhill energy transfer from the higher-energy β exciton (peak B) to the lower-energy α exciton (peak A). By *T* = 60 fs, the amplitude of peak B begins to diminish while peak A grows stronger. At *T* = 150 fs, peak B’s intensity has further decreased, and a faint off-diagonal feature emerges between the two exciton frequencies, indicating the onset of cross-peak formation. By *T* = 300 fs, a pronounced cross peak connecting B (donor) and A (acceptor) is clearly visible. Concomitantly, peak A continues to increase, consistent with population arriving from the depopulating B state. Complete equilibration between the two sites is achieved on the picosecond timescale: At *T* ∼ 600 fs, the cross peaks (labeled C and D in Fig. 1C) and main peaks have stabilized in intensity. The 2DES at *T* = 600 and 1200 fs show that peaks A and B are now connected by well-resolved off-diagonal features, while the ESA band (blue-shaded lobe C between A and B) separates the two bleach signals. These observations confirm that energy has transferred from the β-APC subunit (peak B) to the α subunit (peak A) with a transfer time on the order of a few hundred femtoseconds.

**Fig. 2.**
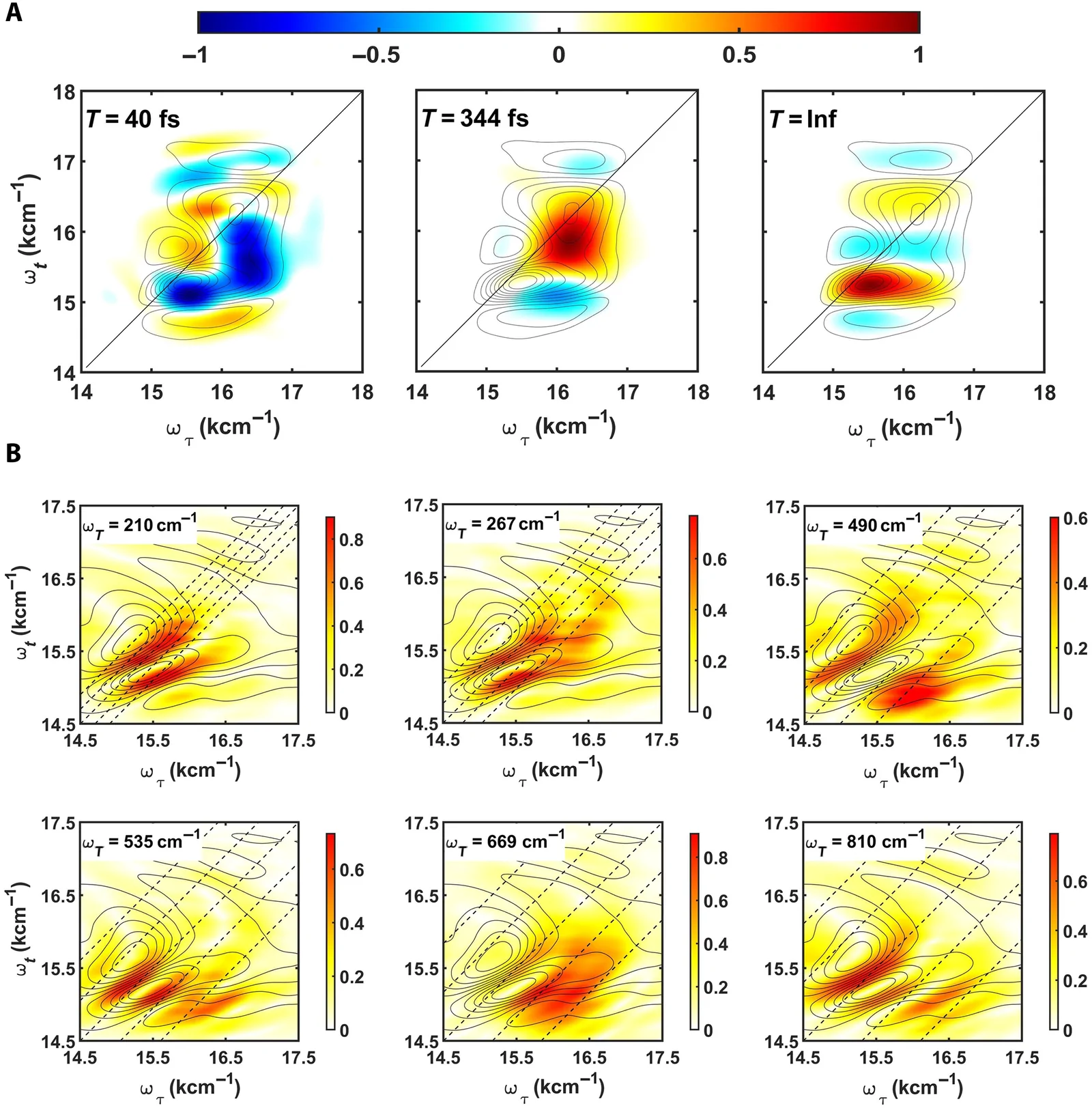
2DDAS and 2D vibrational maps of APC at room temperature. (**A**) Decay-associated spectra (DAS) obtained from global fitting of the 2DES, highlighting dynamics on different timescales. (**B**) Two-dimensional vibrational maps at selected vibrational frequencies, overlaid with 2DES contour lines. Each panel highlights where a particular oscillation frequency is observed in the 2D spectrum. The coherences at ∼210, 267, 490, 535, 669, and 810 cm^−1^ are localized to distinct spectral regions. The vibrational progression is marked by dashed lines.

To further resolve the dynamics, we applied global kinetic analysis, extracting three main decay components (excluding a minor coherent artifact). The decay-associated spectra (Fig. 2A) reveal: (i) a ∼40-fs component dominated by rapid spectral relaxation during pulse overlap; (ii) a ∼344-fs component showing the characteristic pattern of population loss at B and growth at A; and (iii) an “infinite” component reproducing the long-*T* spectrum, representing the static bleach after transfer completes. A weak ∼2-ps component (not shown) also exhibits downhill transfer but with much smaller amplitude.

### Coherences at physiological temperature (300 K)

To uncover the coherences, we removed the population dynamics from selected two-dimensional (2D) signals by subtracting the multiexponential fit, yielding residual oscillatory signals. We then performed Fourier transforms of the residuals (with appropriate windowing) to determine the frequencies of the oscillations (details are described in section S2 in the SI). The Fourier spectra revealed a set of distinct vibrational frequencies: ∼210, 267, 490, 535, 669, and 810 cm^−1^. To visualize where these coherences originate in the 2D spectrum, we constructed coherence maps at selected frequencies. In Fig. 2B (lower panels), the amplitude of the Fourier component at a given vibrational frequency is plotted with the static 2DES contour overlaid for reference. These maps allow us to “map out” which spectral features beat at each frequency. We find that each coherence is localized to specific regions of the 2D spectrum. The ∼210 cm^−1^ coherence appears predominantly on the lower-energy diagonal (around peak A) and its associated cross peak, consistent with a vibrational mode on the α pigment. In contrast, the ∼535 cm^−1^ coherence is strong at off-diagonal positions involving the ESA band, indicating that it may originate from a vibrational wave packet in the excited-state potential or a ground-state bleach modulation involving both pigments. The 669 and 810 cm^−1^ coherences bridge between the bleach of one exciton and the ESA of the other, hinting at a possible vibronic coupling between those states. A definitive assignment, however, demands more stringent validation, and these modes are therefore examined in greater detail in the subsequent sections.

### Temperature-dependent 2DES measurements

**Fig. 3.**
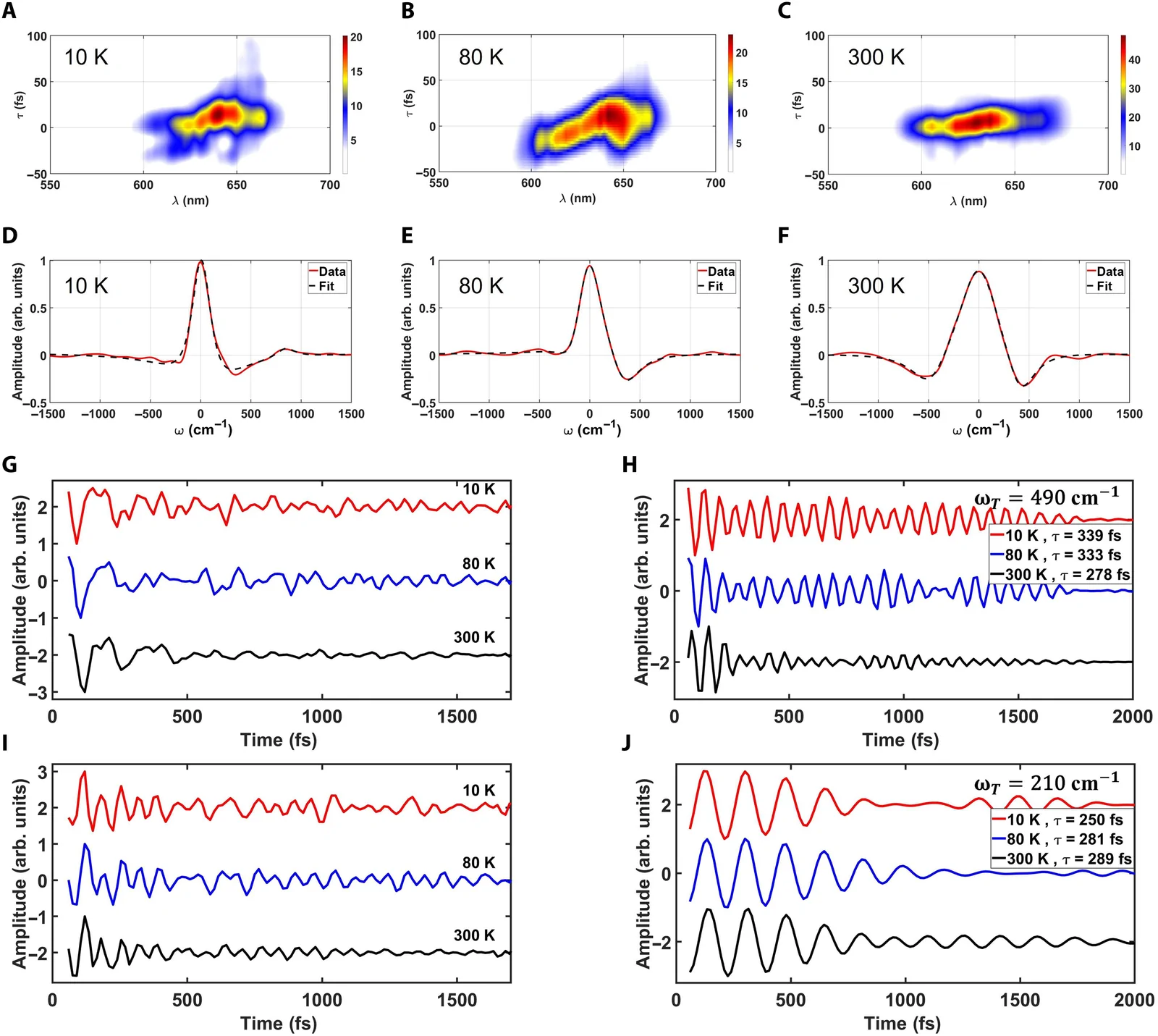
Temperature dependence of electronic dephasing and vibrational coherence in APC. (**A** to **C**) Pseudo-color representation of photon-echo 2D signals at 10, 80, and 300 K. (**D** to **F**) Measured antidiagonal line shapes and Lorentzian fits used to estimate the homogeneous optical dephasing times of the excitonic transitions. (**G** and **I**) Time-domain residual oscillations at peaks A and C mentioned in Fig. 1C (after kinetic subtraction) observed in APC at 10 K (red), 80 K (blue), and 300 K (black), showing the presence of coherent beats. (**H**) Damped oscillatory fits for the 490 cm^−1^ vibrational band at 10, 80, and 300 K, yielding lifetimes of ∼339, ∼333, and ∼278 fs, respectively. (**J**) Fits for the 210 cm^−1^ vibrational band, yielding lifetimes of ∼250, ∼281, and ∼289 fs at 10, 80, and 300 K, respectively.

To further distinguish the relative contributions of vibrational and electronic coherence, we systematically examined the temperature dependence of the coherent dynamics. Lowering the temperature reduces thermal fluctuations and slows environmental dephasing, thereby revealing quantum beats that are otherwise too short-lived at ambient conditions. We acquired 2DES at 10 and 80 K, and subjected them to the same global and coherence analyses as described for 300 K. The full sets of 2DES data at 10 and 80 K, together with extended analyses, are provided in the Supplementary Materials. Photon-echo signals at these three temperatures (Fig. 3, A to C) directly illustrate the contrast and need to acquire for longer detection time window for coherence time at lower temperatures for accurate measurement for dynamics >100 fs. Antidiagonal linewidth analysis yields homogeneous optical dephasing times (*T*_2_) on the order of tens of femtoseconds across all temperatures. The extracted values are 22 fs [L1, full width at half maximum (FWHM) = 475 cm^−1^] and 40 fs (L2, FWHM = 264 cm^−1^) at 10 K, 22 fs (L3, FWHM = 488 cm^−1^) and 32 fs (L4, FWHM = 334 cm^−1^) at 80 K, and 22 fs (L5, FWHM = 482 cm^−1^) and 21 fs (L6, FWHM = 513 cm^−1^) at room temperature (Fig. 3, D to F, and fig. S1B in the SI). In contrast, vibrational coherences display markedly greater resilience. Two representative examples are shown in Fig. 3 (G to J). Because the maxima of the vibrational bands can shift slightly with temperature and detection window, extracting a lifetime at a single fixed frequency can underestimate the coherence lifetime when the selected frequency no longer coincides with the band maximum. We therefore analyzed the representative vibrational bands centered at 490 (±20 cm^−1^) and 210 cm^−1^ (±20 cm^−1^). The 490 cm^−1^ band exhibits fitted lifetimes of ∼339 fs at 10 K, ∼333 fs at 80 K, and ∼278 fs at 300 K. The 210 cm^−1^ band gives comparable lifetimes of ∼250, ∼281, and ∼289 fs at 10, 80, and 300 K, respectively.

### Transient grating measurements

**Fig. 4.**
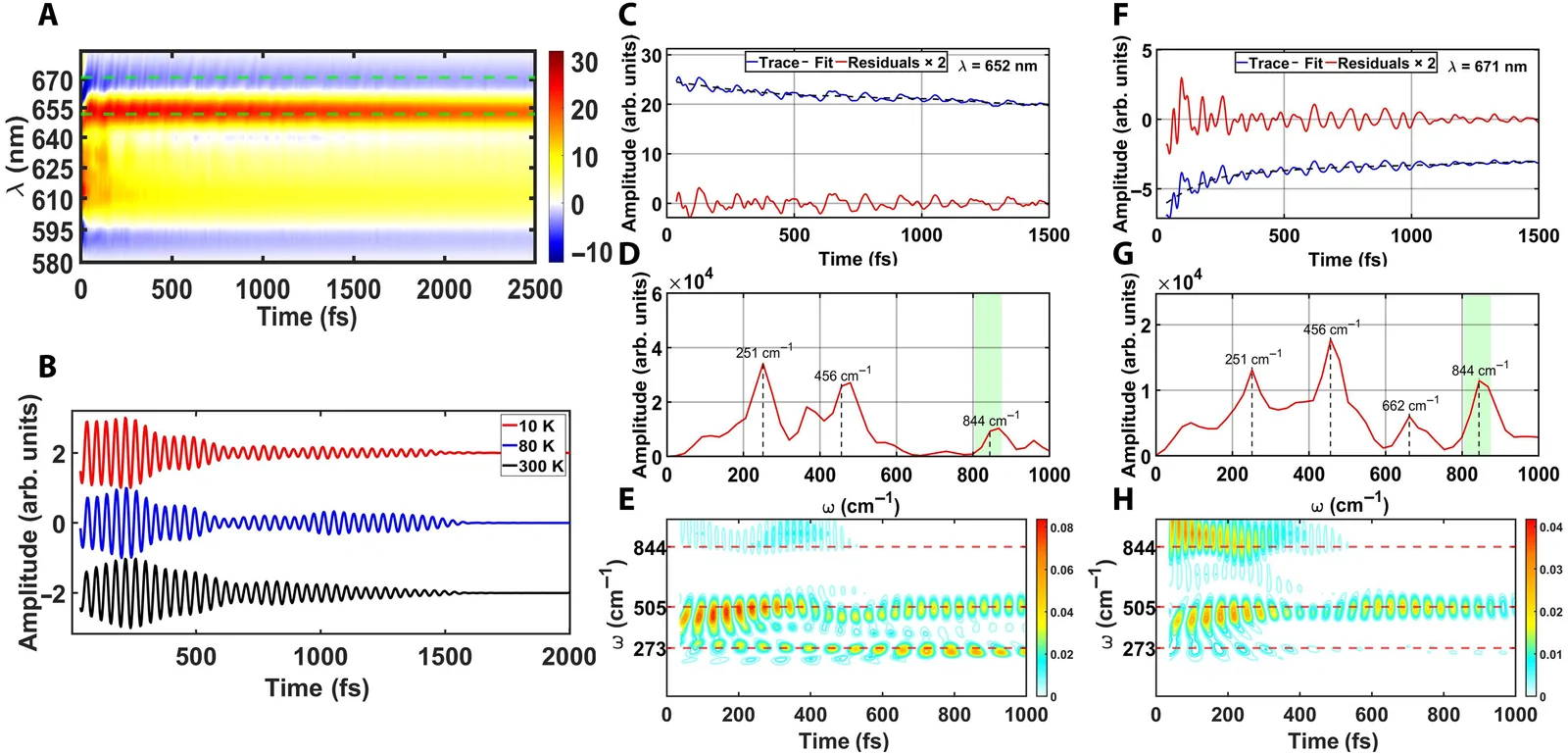
Transient grating analysis of coherent vibrational dynamics in APC. (**A**) Transient grating (TG) spectrogram of APC at 10 K, showing the evolution of the spectrum (580 to 680 nm) as a function of population time. (**B**) Overlaid oscillatory components of the 844 cm^−1^ coherence at 10 K (red), 80 K (blue), and 300 K (black), highlighting its nearly temperature-independent lifetime. (**C** and **F**) TG signals at 10 K for two representative probe wavelengths (652 and 671 nm). Raw data (blue), multiexponential fit (black), and residual oscillations (red) are shown. (**D** and **G**) Fourier transform amplitude spectra of the residuals in (C) and (F). (**E** and **H**) Continuous wavelet spectrograms of the TG residuals, plotting oscillation frequency (vertical axis) versus time (horizontal).

Transient grating (TG) spectroscopy enables the generation of higher-resolution FT maps with reduced data collection requirements, since TG directly records time-domain oscillations at selected probe wavelengths. This streamlined acquisition circumvents the extensive scanning of coherence and detection dimensions necessary in 2DES. Figure 4A presents the spectrally resolved TG signal at 10 K across the 580- to 680-nm region. Individual kinetic traces at each probe wavelength were globally fit with multiexponential functions and the residuals analyzed for coherent oscillations. We highlight two representative detection channels: 652 nm, corresponding to the GSB of the lower-energy exciton (peak A), and 671 nm, positioned in the ESA of the higher-energy exciton (peak B). As shown in Fig. 4 (C and F), both traces reveal pronounced residual oscillations after subtraction of population kinetics. Fourier transforms of these residuals (Fig. 4, D and G) reveal dominant vibrational modes near 250 to 270 cm^−1^ and ∼455 cm^−1^, consistent with the low-frequency wave packets identified in the 2DES analysis. A high-frequency coherence near 844 cm^−1^ is clearly resolved in the ESA region and is also observable in the GSB region, although with a different relative amplitude and spectral clarity. This indicates that the 844 cm^−1^ mode is not exclusive to a single detection channel. Continuous wavelet time-frequency analysis reveals that the 844 cm^−1^ coherence exhibits an initial growth in amplitude up to ∼250 fs, after which it undergoes a gradual decay. Equivalent analyses at 80 and 300 K (see the SI, figs. S8 and S9) confirm the same set of coherences across temperatures. Across temperatures, the 844 cm^−1^ coherence remains observable at 10, 80, and 300 K, whereas the lower-frequency modes show more heterogeneous, mode- and detection window–dependent temperature behavior (Fig. 4B). Complete datasets for *T* = 80 and 300 K are provided in the SI. To consolidate the timescale hierarchy underlying the coherence assignment, Table 1 summarizes the optical dephasing, population-transfer, and vibrational-coherence lifetimes extracted from the linewidth, global kinetic, and wavelet analyses.

**Table 1.** Summary of characteristic timescales governing coherent dynamics in the APC trimer.

Process	10 K	80 K	300 K	Analysis
Optical dephasing (exciton A)	∼40 fs	∼32 fs	∼21 fs	Antidiagonal linewidth
Optical dephasing (exciton B)	∼22 fs	∼22 fs	∼22 fs	Antidiagonal linewidth
Exciton population transfer (B → A)	∼350 fs	∼288 fs	∼344 fs	Global analysis
Low-frequency vibrational coherence (∼210 cm^−1^, averaged)	∼340 fs	∼306 fs	∼306 fs	Wavelet analysis
Mid-frequency vibrational coherence (∼490 cm^−1^, averaged)	∼345 fs	∼432 fs	∼309 fs	Wavelet analysis
High-frequency vibrational coherence (844 cm^−1^)	∼312 fs	∼326 fs	∼395 fs	Wavelet analysis
Electronic coherence lifetime (HEOM)	≤40 fs	≤30 fs	≤20 fs	HEOM simulations

### Theoretical calculations

**Fig. 5.**
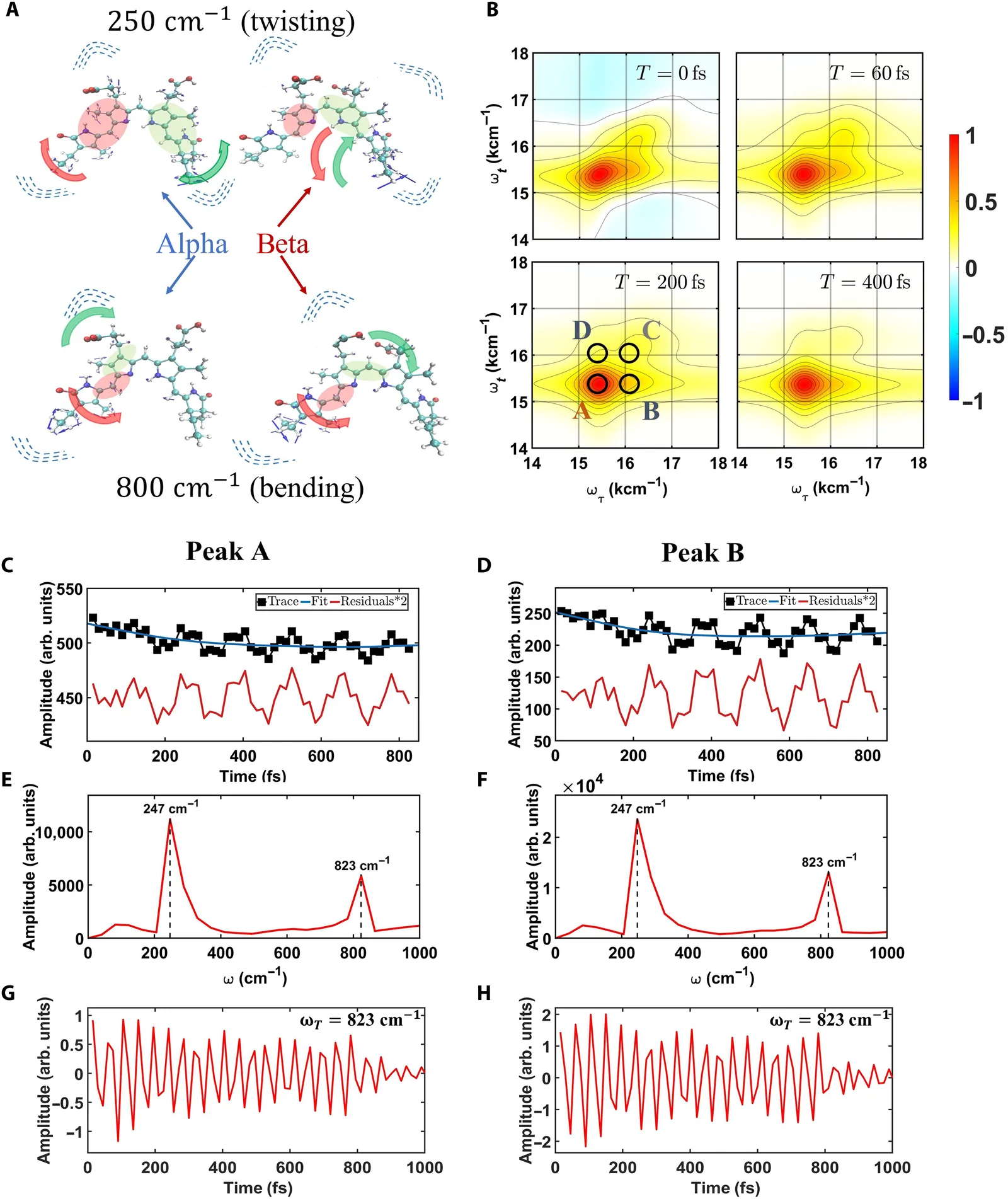
Simulated vibronic dynamics and coherent oscillations in APC. (**A**) Calculated molecular vibrational motions of the 250 and 800 cm^−1^ modes. (**B**) Calculated total real 2DES at waiting times *T* = 0, 60, 200, and 400 fs. (**C** and **D**) Time-resolved traces of selected peaks A and B, with residuals plotted as red solid lines. (**E** and **F**) Fourier transforms of the residuals showing the resolved vibrational modes. (**G** and **H**) Wavelet analysis of the corresponding oscillatory dynamics.

To elucidate the origin of the observed oscillatory dynamics, we performed ab initio calculations to identify the molecular motions underlying each mode. The calculated Raman-active vibrational modes of the APC subunits are presented in Fig. 5, with representative molecular motions illustrated in Fig. 5A. Additional modes and their corresponding vibrational displacements are provided in the SI. Site energies for each cofactor were determined, and excitonic couplings were evaluated using the dipole-dipole approximation. These values were assembled into a system Hamiltonian, with refined parameters detailed in the SI. To account for environmental dissipation, we used a system-bath model, treating protein-induced fluctuations through a linear response framework. Using this approach in conjunction with the HEOM, we calculated the linear absorption spectrum of APC at room temperature. The resulting spectrum, shown as the red trace in Fig. 1B, reproduces the experimental absorption with excellent agreement.

With the refined Hamiltonian, we further simulated 2D electronic spectra at waiting times of 0, 60, 200, and 400 fs. As shown in Fig. 5B, the simulated 2DES reproduces both the main diagonal features and the cross peaks observed experimentally (Fig. 1C), confirming the robustness of our model. To track population and coherence dynamics, we analyzed the time-dependent signals of two diagonal (A and B) and two cross peaks, which have been labeled at *T* = 200 fs. The time traces of peaks A and B are shown in Fig. 5 (C and D), where raw data (black dots) are fitted by simulated curves (blue). Residuals from these fits (red) reveal oscillatory behavior, and Fourier transformation of the residuals yields vibrational frequencies at 247 and 823 cm^−1^ (Fig. 5, E and F). These values are in close correspondence with the experimentally observed modes and the theoretically assigned Raman modes, validating the accuracy of the calculations. We further performed wavelet analysis and show the time-resolved coherence at 823 cm^−1^ in Fig. 5 (G and H), respectively. On the basis of our calculations, we show that the lifetime of the coherence associated with the vibrational mode could last for more than 800 fs, even at room temperature. Compared to the experimental observation in Fig. 4, we conclude that our theoretical results excellently agree to the experimentally revealed timescales of coherence for the vibrational mode at 844 cm^−1^, which demonstrates the validity of our model and refined parameters.

However, current theoretical treatments have proved inadequate in unambiguously distinguishing the precise origin of the observed coherence, whether vibrational or vibronic in character. To overcome this, we calculated population dynamics using a refined model, with the full results given in section S15 of the Supplementary Materials. These simulations show that the electronic coherence near 790 cm^−1^ is strongly mixed with the assigned vibrational component, producing a beat that lasts several hundred femtoseconds before disappearing around 800 fs. We then varied the vibrational frequency (700 to 900 cm^−1^; Supplementary Materials, section S15) and observed a distinct separation between electronic and vibrational coherences whenever their frequencies diverged. Even in the resonant case (where the vibrational mode matches the α - β energy gap) no enhancement or stabilization of electronic coherence occurs; instead, both amplitude and lifetime change smoothly with frequency. Additional calculations with different system-bath coupling strengths similarly show that oscillation lifetimes extend or shorten monotonically with coupling strength. Together, these results demonstrate that molecular vibrations cannot significantly prolong electronic coherence between the α and β pigments, even under resonance.

### APC monomer study

As a control, we performed 2DES and TG measurements on isolated APC monomers (single αβ chromophore pairs, without interunit coupling, figs. S20 and S21). The monomer spectra show the same oscillatory coherence patterns found in the trimer, confirming their intramolecular origin. In particular, we observe a distinct vibrational coherence at ∼840 cm^−1^ in the monomer, closely matching the high-frequency beat in the trimer. The strong agreement between the monomer and trimer results, both experimentally and theoretically (fig. S22), supports our assignment.

## DISCUSSION

Our combined spectroscopic and theoretical analysis provides strong evidence, within the APC complex investigated here, that the long-lived oscillatory signals are predominantly vibrational in origin. By integrating temperature-dependent 2DES, TG spectroscopy and nonperturbative quantum dynamical simulations, we show that electronic coherence decays substantially faster than the observed excitation-energy transfer process under the investigated conditions. Although the excitonic states in APC are delocalized across the α and β chromophore subunits, the optical dephasing associated with the excitonic transitions occurs within less than a vibrational period under physiological conditions. The corresponding homogeneous optical dephasing times are ∼20 to 22 fs at room temperature and no more than 40 fs even at cryogenic temperatures, far shorter than the roughly 344-fs energy-transfer step. Together with the waiting-time analyses and the temperature dependence of the oscillatory signals, this supports the conclusion that any purely electronic coherence is unlikely to persist long enough to influence energy transfer under physiological conditions [although the antidiagonal linewidth does not directly measure zero-quantum excited-state coherence (*50*), it constrains the strength and timescale of the bath-induced energy-gap fluctuations that also drive excitonic dephasing]. Therefore, in the Markovian limit and in the absence of long-range spatially correlated bath fluctuations, the homogeneous linewidth provides a physically meaningful upper-bound/consistency check on possible electronic coherence lifetimes. Energy flow in APC therefore proceeds through incoherent downhill relaxation, rather than through wave-like or phase-coherent transport. In contrast to this rapid electronic decoherence, vibrational coherences remain notably robust. Low-frequency modes display temperature-dependent lifetimes that reflect their coupling to the protein environment. Most notably, a high-frequency mode near 840 cm^−1^ shows a characteristic rise in amplitude during the first few hundred femtoseconds and then decays only on the timescale of nuclear motion. Its lifetime is essentially unchanged from 10 to 300 K, demonstrating that it originates from a strongly localized intramolecular vibration rather than an electronic superposition. This persistence, together with its resonance with the excitonic energy gap, clarifies why vibrational oscillations can mimic exciton-frequency beats without supporting long-lived electronic coherence. Our simulations further confirm that vibronic resonance does not extend electronic phase memory: Even under resonant conditions, electronic coherence collapses within a few tens of femtoseconds.

Recent work has proposed that long-lived coherence in APC may arise from exciton-vibrational phase synchronization, where resonant collective modes become protected against environmental dissipation (*47*). In this framework, selective damping of antisymmetric modes leads to sustained coherent dynamics and has been interpreted as evidence of coherence protection mechanisms. In contrast, our results show that comparable long-lived oscillatory features are also observed in the APC monomer, where the interpigment excitonic coupling is only 9.64 cm^−1^ and can therefore be considered negligible. This provides strong evidence that these oscillations do not require dimer-specific coherence protection mechanisms and instead are consistent with predominantly intramolecular vibrational coherences that persist independently of excitonic delocalization. We note, however, that the APC monomer still contains an α-β chromophore pair and therefore cannot, by itself, exclude all possible intramonomer vibronic mixing. Rather, the observation of similar oscillatory features in both monomer and trimer shows that the long-lived beatings do not require intermonomer coupling or collective trimer-level delocalization. Because the calculated excitonic coupling within the isolated monomer is small (9.64 cm^−1^), the persistence of these oscillations in the monomer is more consistent with a predominantly local vibrational origin. This observation is not consistent with the possibility that long-lived oscillations arise from interchromophore coupling or from any collective delocalized mechanism. Instead, these oscillations reflect intrinsic pigment vibrations and persist independently of excitonic architecture. The monomer-trimer comparison thus provides strong supporting evidence that vibrational coherence is a property of the chromophores themselves, not a manifestation of multichromophoric quantum coherence.

Although the present study focuses on the APC trimer, the methodology developed here is generally applicable to a broad class of excitonic systems in which the origin of coherent oscillations remains under debate. Temperature-dependent 2D electronic spectroscopy provides a particularly powerful framework because electronic coherence, vibronic coherence, and purely vibrational coherence exhibit fundamentally different responses to environmental fluctuations. Electronic coherences are expected to shorten markedly with increasing temperature owing to enhanced system-bath interactions, whereas local vibrational wave packets often remain comparatively robust. By directly comparing coherence lifetimes with the corresponding electronic dephasing and population-transfer timescales, together with microscopic modeling, this approach provides a practical route for distinguishing the physical origin of oscillatory signals without relying solely on polarization-selective measurements or coherence-map analysis. We therefore anticipate that the same methodology can be readily extended to other photosynthetic antenna complexes, reaction centers, and artificial excitonic materials where the assignment of long-lived coherences remains an open question.

Together, these findings situate APC within an emerging consensus across photosynthetic complexes such as FMO and photosystem II, where oscillatory signals initially attributed to electronic quantum beats have been reassigned to vibrational motion (*51*). In this context, it is instructive to compare APC with other photosynthetic complexes such as FMO, LHCII, and reaction centers, which occupy markedly different excitonic parameter regimes. Despite these differences in energy gaps and coupling strengths, our results show that the electronic coherence lifetime remains consistently ultrafast across systems. This cross-system comparison highlights a parameter-space invariant behavior and illustrates that the persistence of electronic coherence is not enhanced even in near-resonant regimes. Together with temperature-resolved measurements and theory-experiment consistency, this study provides a broader framework for understanding the general role of coherence in photosynthetic energy transfer. Vibronic mixing can transiently imprint exciton-gap frequencies at very low temperatures, but under physiological conditions, electronic coherence decays almost immediately, leaving only vibrational wave packets detectable. APC offers a particularly clear example, owing to the fortuitous overlap between excitonic splitting and pigment vibrational frequency, which demonstrates explicitly how vibrational coherences can masquerade as electronic phenomena.

The broader implications of this work extend to the conceptual foundations of quantum biology. Although vibrational coherences may subtly influence relaxation landscapes or serve as probes of protein environments, they may affect energy transfer in APC. The persistence of high-frequency vibrations long after population equilibration illustrates their spectroscopic, rather than functional, role. Photosynthetic systems achieve high efficiency not through sustained quantum coherence but through optimized pigment arrangements, energetically downhill pathways, and appropriately tuned interactions with their environment. Looking ahead, our results establish a complementary experimental and theoretical framework for distinguishing vibrational from electronic coherence in biological light-harvesting systems. The combination of temperature variation, architectural comparison (such as monomer versus trimer), and quantitative quantum dynamics should serve as a template for future studies across diverse pigment-protein complexes. By providing a clearer assignment of the coherence origin in APC, this work helps steer quantum biology toward a mechanistic, experimentally anchored understanding of exciton-vibration interactions and their true role in nature’s light-harvesting machinery. While these findings do not constitute a universal resolution of the role of coherence in photosynthetic energy transfer, they provide strong evidence that, within the APC complex investigated here, electronic coherence is short-lived and unlikely to make a significant functional contribution under physiological conditions.

## MATERIALS AND METHODS

### Sample preparation

Cross-Linked Allophycocyanin (CL-APC; item no. 97052) was purchased from Crystal BioSystems and used without further modification. The APC complex was initially dissolved in the solution (20 mM PB, 2 mM EDTA) and diluted to the maximum of optical density 0.25 (absorption spectrum) at wavelength of 650 nm. To obtain a monomer sample, the trimeric APC sample has been further dissolved in urea (8 M concentration). The purification of APC monomer was confirmed by measuring absorption spectrum. The sample (monomeric APC and crosslinked APC trimer) was transferred into a 1-mm quartz cuvette for measurements conducted at room temperature. The cuvette was mounted on a custom-built shaker system with controllable X-Y motion, allowing precise adjustment of shaking speed via a regulated power supply. In addition, a home-constructed XYZ delay stage enabled fine tuning of the laser focus on the sample, facilitating optimization of beam size and spatial positioning for TA, TG, and 2DES measurements, while minimizing scattering to enhance the signal-to-noise ratio. The APC trimer sample was mixed thoroughly with glycerol in a 1:4 volume ratio and loaded into a custom sample cell. This assembly was then placed in a closed-cycle cryostat (ARS system, helium-cooled) for temperature-controlled measurements at 10 and 80 K.

### 2D and TG electronic measurements with experimental conditions

The details of the experimental setup follow previous reports from our group (*52*, *53*). In brief, 2D electronic spectra were recorded using a diffractive optics-based, all-reflective 2D spectrometer providing phase stability of λ/160. Excitation pulses were generated by a home-built nonlinear optical parametric amplifier, pumped by a commercial femtosecond laser system (Spectra Physics, Newport). The pulses were compressed to ∼10 fs using a combination of a deformable mirror (OKO Technologies, 19-channel) and a fused silica prism pair. Their temporal profile was characterized by frequency-resolved optical gating (FROG), with the traces analyzed using the commercial package FROG3 (Femtosecond Technologies). The resulting broadband spectrum had a ∼100-nm FWHM, centered at 620 nm, sufficient to cover the main electronic transitions into the lowest excited states. For 2DES measurements, three beams were focused onto the sample with a spot size of ∼130 μm, generating a photon-echo signal along the phase-matching direction. The emitted signal was collected using a Sciencetech 9055F spectrometer coupled to a charge-coupled device linear array detector (Entwicklungsbüro Stresing). TG spectra were recorded at multiple waiting times *T* by scanning the population delay from −200 fs to 3 ps with 3-fs steps. At each delay point, 200 individual spectra were averaged to improve the signal-to-noise ratio. For all experiments, the excitation pulse energy was attenuated to ∼10 nJ at a 1-kHz repetition rate. Phasing of the TG data was achieved using the “invariant theorem” procedure described previously (*54*).

### Model Hamiltonian and parameters

For an open quantum system, the total Hamiltonian can be generally expressed as

$$H=H_{S}+H_{B}+H_{\text{SB}}$$ (1)

where $H_{S}$ denotes the Hamiltonian of the system of interest, $H_{B}$ represents the Hamiltonian of the surrounding environment (bath), and $H_{\text{SB}}$ captures the interaction between the system and the bath. To model excitation energy transfer in the APC light-harvesting complex, we consider a vibronic Hamiltonian that explicitly includes both the electronic and vibrational degrees of freedom of the chromophores. The APC trimer contains two primary pigment types, labeled $\alpha_{84}$ and $\beta_{84}$, each capable of supporting multiple vibrationally excited electronic states. The system Hamiltonian can be written as

$$\begin{array}{c} H_{S}=\sum_{i=1}^{n}\left(|a_{i}\rangle \left(\epsilon_{a}+h_{i}\right)\langle a_{i}|+|b_{i}\rangle \left(\epsilon_{b}+h_{i}^{\prime }\right)\langle b_{i}|+|a_{i}b_{i}\rangle \left(\epsilon_{a}+\epsilon_{b}+h_{i}+h_{i}^{\prime }\right)\langle a_{i}b_{i}|\right)\\ +\sum_{m\neq n}|a_{m}\rangle V_{\mathrm{mn}}\langle b_{n}| \end{array}$$ (2)

where $|a_{i}\rangle$ and $|b_{i}\rangle$ denote the vibronic basis states corresponding to the $\alpha_{84}$ and $\beta_{84}$ pigments, respectively. Here, $|a_{i}b_{i}\rangle$ denotes the double-excited state, where both the electronic states $a_{i}$ and $b_{i}$ are simultaneously excited. The energies ϵ*_a_* = 15,300 cm^−1^ and ϵ*_b_* = 16,060 cm^−1^ represent the electronic site energies of the $\alpha_{84}$ and $\beta_{84}$ pigments, derived from experimental spectroscopic data. The term *V_mn_* = −157 cm^−1^ captures the excitonic coupling between vibronic states located on different pigments and facilitates population transfer and excitonic coherence between the two pigments.

The vibrational structure of each site is included via the site-local vibrational Hamiltonian, which is given as

$$h_{i}=\sum_{j=1}^{2}\frac{1}{2}\hbar \Omega_{j}\left(\alpha_{i,j}^{†}\alpha_{i,j}+\frac{1}{2}\right)+\kappa_{j}Q_{j}$$ (3)

$$h_{i}^{\prime }=\sum_{j=1}^{2}\frac{1}{2}\hbar \Omega_{j}\left(\alpha_{i,j}^{†}\alpha_{i,j}+\frac{1}{2}\right)+\kappa_{j}^{\prime }Q_{j}$$ (4)

where Ω_1_ = 250 cm^−1^ and Ω_2_ = 800 cm^−1^ are the frequency of a high-energy intramolecular vibrational mode, and α^†^ (α) are the bosonic creation (annihilation) operators of the vibrational quanta. *Q* is the vibrational reaction coordinate, and the site-specific coupling strength κ*_j_* quantifies the vibronic coupling at each pigment site. This term modulates the electronic site energy as a function of nuclear motion, enabling electronic-vibrational state mixing. The index *i* runs over *n* vibrational states per site, representing quantized vibrational excitations built on the electronic ground and excited states. Together, this vibronic Hamiltonian provides a realistic and flexible model that captures the essential physics of APC pigment-protein interactions. It serves as a foundation for simulating excitation energy transport using nonperturbative approaches such as the HEOM, enabling the study of quantum coherence, energy relaxation, and environmental effects in photosynthetic systems. The bath Hamiltonian is described as a set of harmonic oscillators, which shows the form

$$H_{B}=\sum_{i=1}^{N}\left(\frac{p_{i}^{2}}{2m_{i}}+\frac{1}{2}m_{i}\omega_{i}^{2}x_{i}^{2}\right)$$ (5)

where *N* is the total number of bath modes. The $x_{i}$ and $p_{i}$ are the mass-weighted position and momentum of *i*th harmonic oscillator bath mode with frequency $\omega_{i}$. The interaction term $H_{\text{SB}}=\sum_{i}K_{i}\Phi_{i}\left(x\right)$ characterizes the coupling between the system and the bath. Assuming a linear coupling of the system to the bath coordinates, the interaction can be reformulated as $H_{\text{SB}}=\sum_{i}K_{i}c_{i}x_{i}$, where $c_{i}$ denotes the coupling constants and $x_{i}$ represents the bath coordinates. The distribution of bath modes can be expressed as the spectral density, which shows the form $J\left(\omega \right)=\pi \sum_{i}\frac{c_{i}^{2}}{2m_{i}\omega_{i}}\delta \left(\omega -\omega_{i}\right)$.

## Supplementary Materials

### This PDF file includes:

Supplementary Text Figs. S1 to S25 Table S1 References
