# Supplementary material for: Quantum coherent dynamics in allophycocyanin trimer complex
Source: Sci Adv. 2026 Sep 16;12(38):eaef1022. doi: 10.1126/sciadv.aef1022 (PMC13580574; doi:10.1126/sciadv.aef1022)
Supplement: Supplementary file 1 [file sciadv.aef1022_sm.pdf]

Science Advances
AAAS

Supplementary Materials for

Quantum coherent dynamics in allophycocyanin trimer complex

Tianrui Chen *et al.*

Corresponding author: Fulu Zheng, zhengfulu@nbu.edu.cn; R. J. Dwayne Miller, dwayne.miller@utoronto.ca; Ajay Jha, ajay.jha@rfi.ac.uk; Hong-Guang Duan, duanhongguang@nbu.edu.cn

Sci. Adv. **12**, eaef1022 (2026)
DOI: 10.1126/sciadv.aef1022

This PDF file includes:

Supplementary Text
Figs. S1 to S25
Table S1
References

In this Supplementary Material, we first analyze the anti-diagonal bandwidths and the corresponding electronic dephasing lifetimes at different temperatures. We then present the measured 2DES of the allophycocyanin trimer complex and describe the refinement and smoothing procedures used for data processing. The Fourier-transform and wavelet-analysis results are presented together with the temperature-dependent 2D vibrational maps. We also describe the *ab initio* calculations of site energies and electronic couplings, the hierarchical equations of motion (HEOM), the calculated 2DES, and the transient-grating spectroscopy results.

S1. FITTING PROCEDURE OF THE ANTI-DIAGONAL BANDWIDTH

In this section, we describe the procedure used to estimate the homogeneous optical dephasing times from the anti-diagonal linewidths of the 2D spectra. The anti-diagonal linewidth provides an approximate measure of the homogeneous optical dephasing time of the excitonic transitions. Because the spectra at zero waiting time were affected by strong pulse-overlap contributions, we used the 2DES at a waiting time of 30 fs for this analysis. We estimated the homogeneous optical dephasing times by measuring the anti-diagonal bandwidths along line cuts L1–L6 in the 2D spectra (Fig. S1(a)). The bandwidth of each line cut was obtained by fitting the signal to a Lorentzian lineshape

$$y = y_0 + \sum_i \left(\frac{A_i}{1 + 4\left(\frac{(x - x_i)}{w_i}\right)^2}\right). \tag{S1}$$

We extracted the anti-diagonal profiles along the line cuts L1–L6 marked in Fig. S1(a). The retrieved profiles are shown as red solid lines in Fig. S1(b). We fitted the profiles with Lorentzian lineshape functions, and the converged fits are plotted as black dashed lines. Fitting was performed using Curve Fitting Toolbox in MATLAB R2022b. Each fitted Lorentzian profile yields a full width at half maximum (FWHM), w_i. The electronic dephasing time was calculated from the fitted bandwidth as $\tau_{\text{hom}} = (\pi c w_i)^{-1}$, where c is the speed of light. The fitted linewidths and corresponding dephasing times are listed in Table S1.

TABLE S1. Parameters of Lorentzian fits for the anti-diagonal line cuts L1–L6 shown in Fig. S1.

	L1	L2	L3	L4	L5	L6
$w_i(kcm^{-1})$	0.475	0.264	0.488	0.334	0.482	0.513
τ_{hom} (fs)	22.33	40.19	21.74	31.76	22.02	20.68

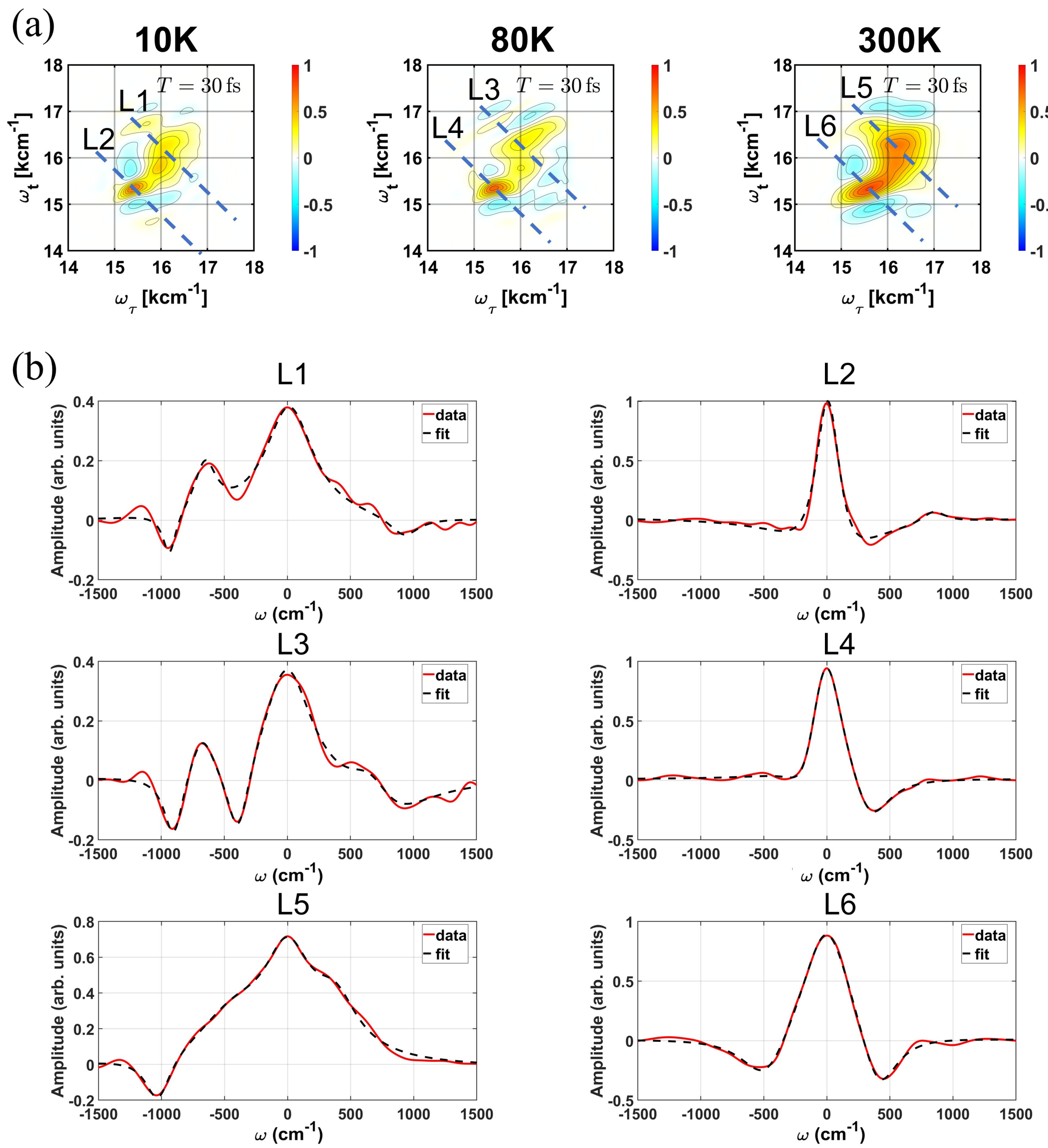

FIG. S1. (a) Two-dimensional electronic spectra at 10, 80, and 300 K, with dashed lines marking the anti-diagonal line cuts L1–L6 used for linewidth analysis. (b) Measured anti-diagonal profiles and Lorentzian fits for the corresponding line cuts L1–L6, used to estimate the homogeneous optical dephasing times.

S2. TUKEY-WINDOW FOURIER TRANSFORM

In this section, we describe the Fourier-transform procedure using a Tukey window. To suppress high-frequency noise, we applied Fourier-domain filtering and isolated each frequency region of interest using a Tukey window of the form

$$\omega(n) = \begin{cases} 1, & 0 \leq |n| \leq \alpha \frac{N}{2} \\ \frac{1}{2}\left(1 + \cos\left[\frac{\pi(n - \alpha\frac{N}{2})}{(1-\alpha)\frac{N}{2}}\right]\right), & \alpha\frac{N}{2} \leq |n| \leq \frac{N}{2}. \end{cases} \tag{S2}$$

Because of its flat top, this window preserves the amplitudes of the Fourier components of interest over a broader frequency range than a cosine or Gaussian window while limiting artifacts from a sharp bandpass filter. In this work, we used $\alpha = 0.15$ and retained frequencies up to 900 cm^{-1}.

S3. COHERENT DYNAMICS OF PEAKS B AND D AT ROOM TEMPERATURE

Having analyzed peaks A and C in the main text, we next analyzed peaks B and D. Following the same protocol, amplitudes at selected spectral coordinates were extracted, and the corresponding time-resolved traces are plotted as blue solid lines in Fig. S2(a) and Fig. S2(d), respectively. The population kinetics were modeled by a global fit and subtracted; the remaining residual oscillations are shown as red solid lines in the same panels. Wavelet analysis was then used to track the time-resolved oscillatory dynamics, and the corresponding spectrograms are presented in Fig. S2(b) and Fig. S2(e). Because the peak positions of the vibrational bands varied slightly across spectral coordinates, we assigned the dominant low- and mid-frequency components to bands centered near 210 and 490 cm^{-1}, respectively. In Fig. S2(b), these bands are highlighted for peak B. Damped-oscillation fits yielded lifetimes of ∼309 fs for the 222 cm^{-1} component and ∼397 fs for the 505 cm^{-1} component. For peak D, the corresponding bands are shown in Fig. S2(e), and the fits yielded lifetimes of ∼319 fs for the 202 cm^{-1} component and ∼251 fs for the 505 cm^{-1} component, as summarized in Fig. S2(f).

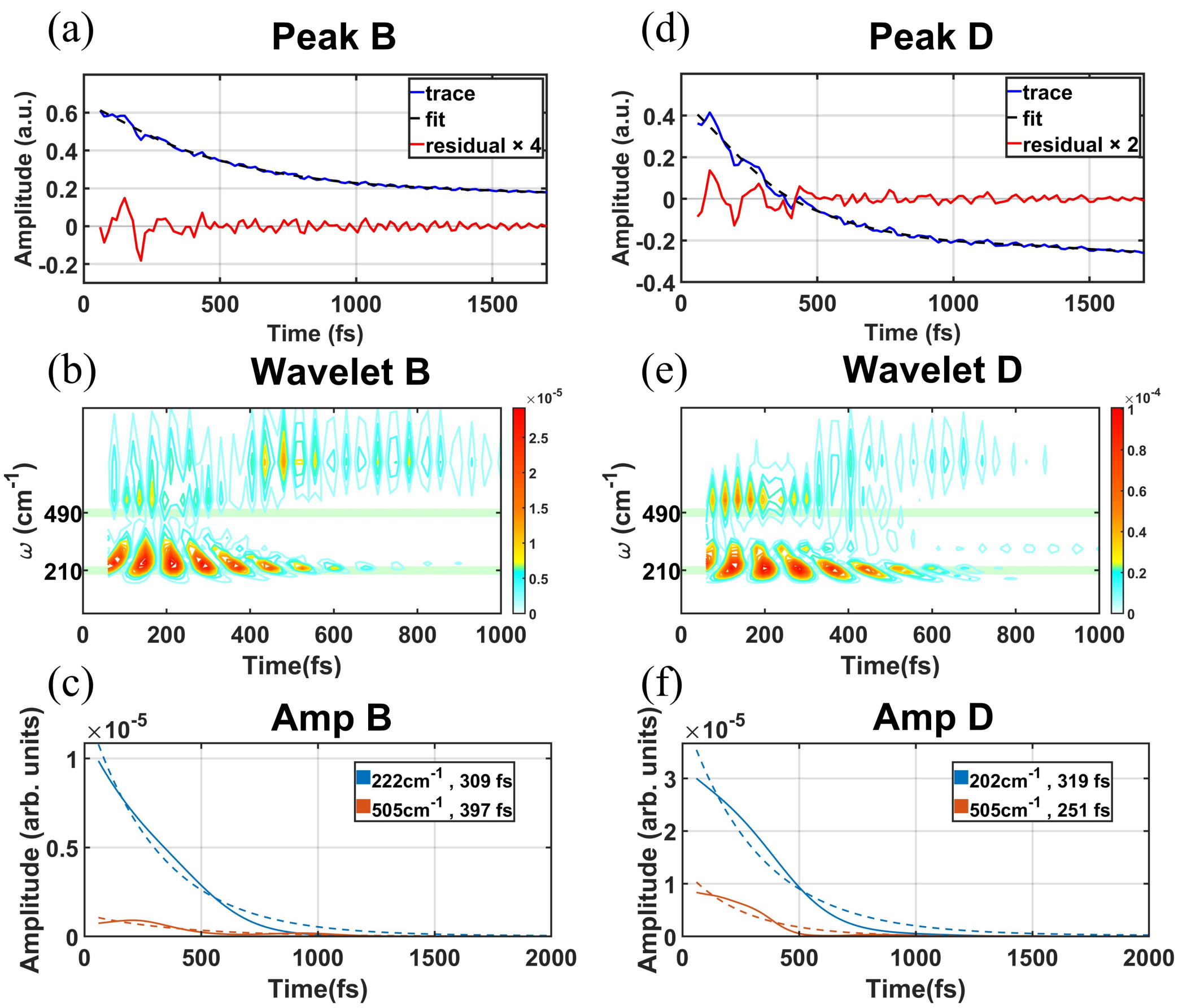

FIG. S2. The time-resolved traces of peaks B and D are shown in (a) and (d). Wavelet analysis of the residuals is shown in (b) and (e), respectively, with the dominant vibrational bands centered near 210 and 490 cm^{-1}. Time-resolved amplitudes after damped-oscillation fitting are shown in (c) and (f). The measurement temperature is 300 K.

S4. TWO-DIMENSIONAL ELECTRONIC SPECTRA AT 10 K AND 80 K

In this section, we present the measured 2D electronic spectra of the allophycocyanin trimer complex at 10 and 80 K. Figure S3(a) shows the real parts of the spectra measured at 10 K at waiting times of 30, 60, 150, 300, 600, and 1200 fs. Positive and negative amplitudes correspond to ground-state bleach (GSB) and excited-state absorption (ESA), respectively. Figure S3(b) shows the corresponding spectra measured at 80 K.

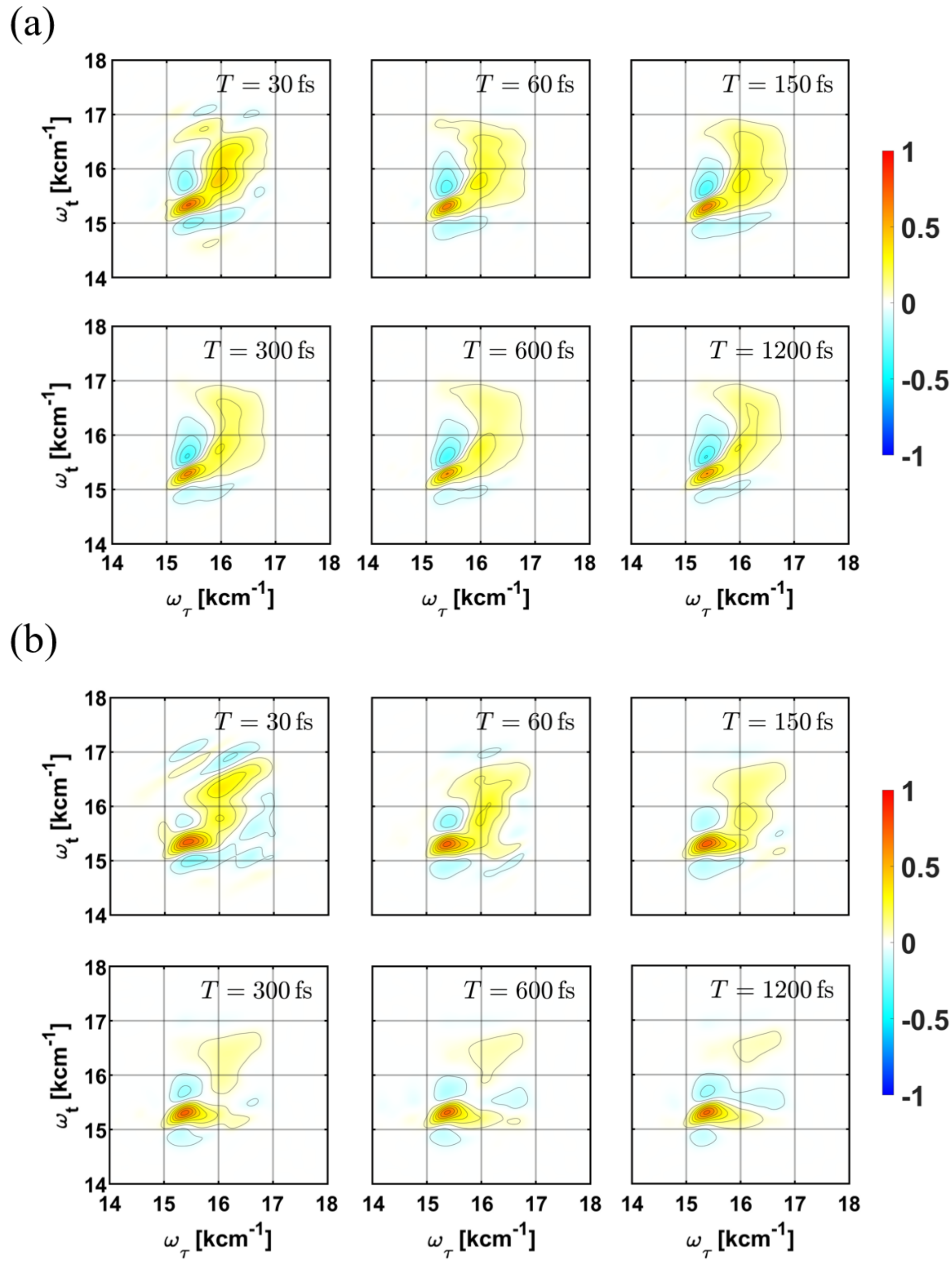

FIG. S3. Real parts of the 2D electronic spectra at 10 K (a) and 80 K (b) with selected waiting times.

S5. GLOBAL FITTING APPROACH AND DECAY-ASSOCIATED SPECTRA

In this section, we describe the global fitting analysis of the 2D spectra. Multidimensional global fitting of the experimental 2D spectral arrays was performed using a previously developed algorithm (55). A sequence of 2D spectra was assembled into the three-dimensional (3D) array $\mathrm{S}(\omega_\tau, \mathrm{T}, \omega_\mathrm{t})$. This array was decomposed into a sum of

2D decay-associated spectra $A_i(\omega_\tau, \omega_t)$, each associated with an exponential lifetime τ_i, according to

$$S(\omega_\tau, T, \omega_t) = \sum_{i=1}^{N} A_i(\omega_\tau, \omega_t) e^{-T/\tau_i}. \tag{S3}$$

The resulting 2DDAS identify spectral coordinates with decaying or rising amplitudes in the 2DES. Positive (red) amplitudes indicate exponential decay with the resolved timescale τ, whereas negative (blue) amplitudes indicate exponential growth with the same timescale. The measurement window extended to 2 ps with a time step of 15 fs. Three exponential functions were used to fit the kinetics in the 3D data set. At 10 K, the analysis resolved components with time constants of 11 and 350 fs, together with an Inf component (Fig. S4(a)). At 80 K, components of 16 and 288 fs and an Inf component were obtained (Fig. S4(b)).

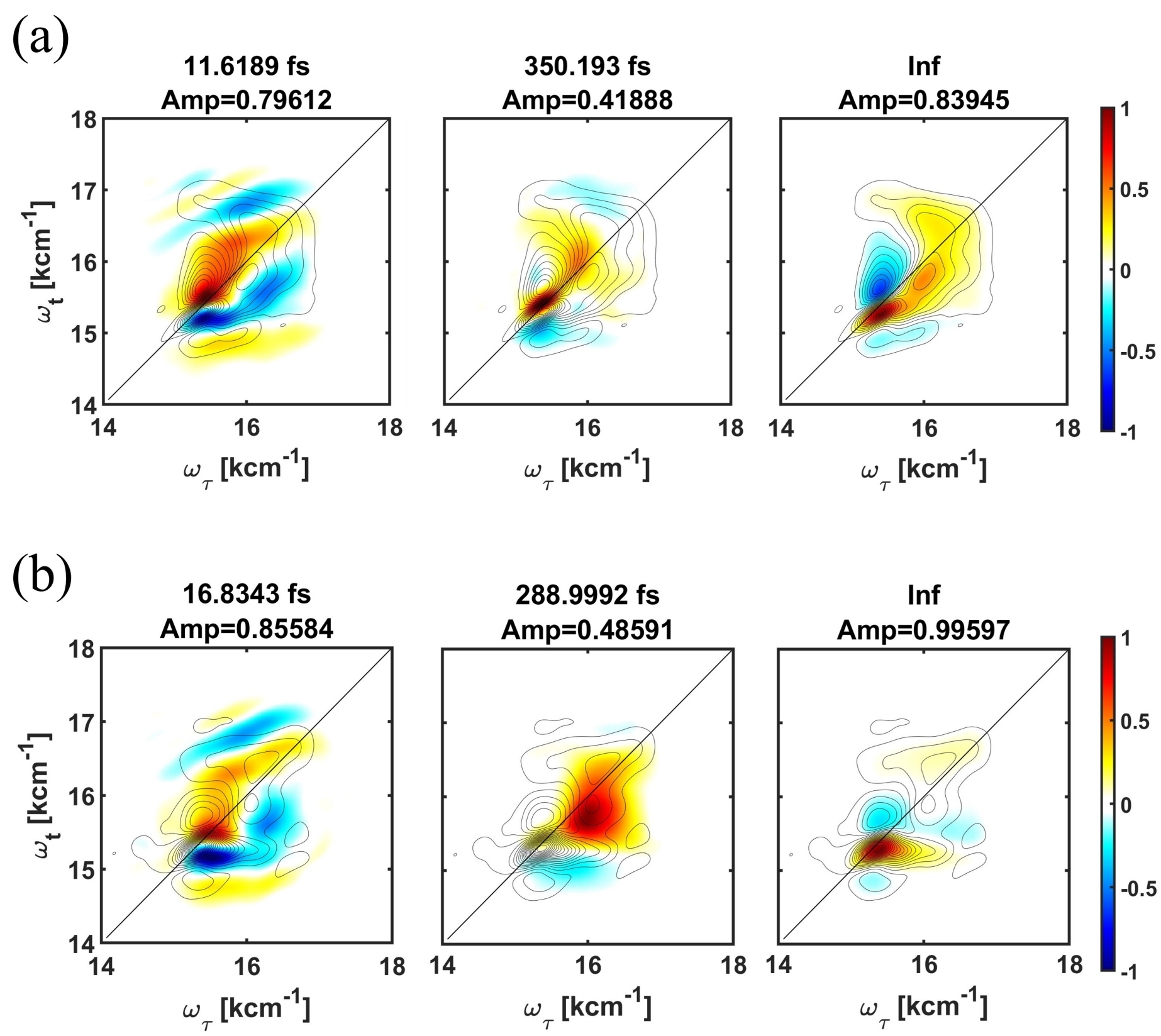

FIG. S4. Two-dimensional decay-associated spectra and the corresponding resolved time constants at (a) 10 K and (b) 80 K.

S6. 2D VIBRATIONAL MAPS

In this section, we describe the generation of the 2D vibrational maps. The 3D residuals were obtained by removing the kinetic components identified by global fitting of the rephasing 2DES. A Fourier transform was then performed along the waiting-time axis, T, to obtain oscillation amplitudes along the excitation and detection frequency axes. The resulting maps at 10 and 80 K are shown in Fig. S5(a) and Fig. S5(b), respectively. Oscillatory features were resolved at 210, 268, 490, 535, 669, and 810 cm^{-1}, and the vibrational progression is indicated by dashed lines. For comparison, the 2DES at a representative waiting time of $T = 700$ fs is displayed alongside each map to show the spectral locations of the oscillatory amplitudes.

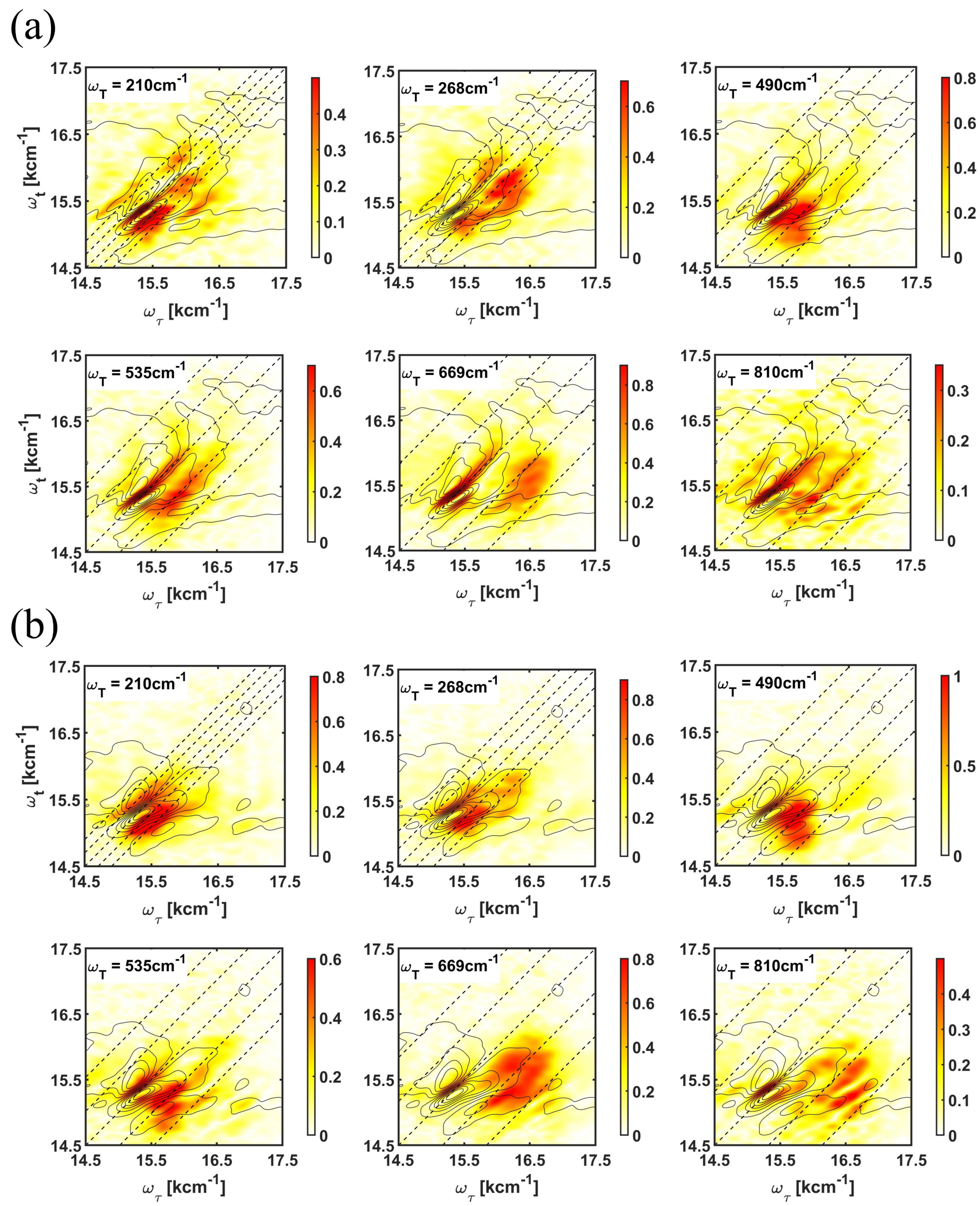

FIG. S5. Two-dimensional vibrational maps at (a) 10 K and (b) 80 K overlaid with 2DES contour lines. The vibrational progression is indicated by dashed lines.

S7. TEMPERATURE-DEPENDENT ELECTRONIC AND VIBRATIONAL COHERENCES OF PEAKS B AND D

Residuals for peaks B and D at 10, 80, and 300 K are summarized in Fig. S6(a) and Fig. S6(e). Fourier-domain filtering was used to remove high-frequency noise. The lifetime analysis tracked bands centered at 210 cm^{-1} ($\pm$20 cm^{-1}) and 490 cm^{-1} ($\pm$20 cm^{-1}), rather than a single fixed frequency point. Figures S6(b) and S6(f) show the corresponding Fourier spectra and band selections. For peak B, the 210 cm^{-1} band yielded lifetimes of 390, 313, and 309 fs at 10, 80, and 300 K, respectively, whereas the 490 cm^{-1} band yielded lifetimes of 294, 480, and 397 fs. For peak D, the corresponding lifetimes were 381, 325, and 319 fs for the 210 cm^{-1} band and 401, 483, and 251 fs for the 490 cm^{-1} band.

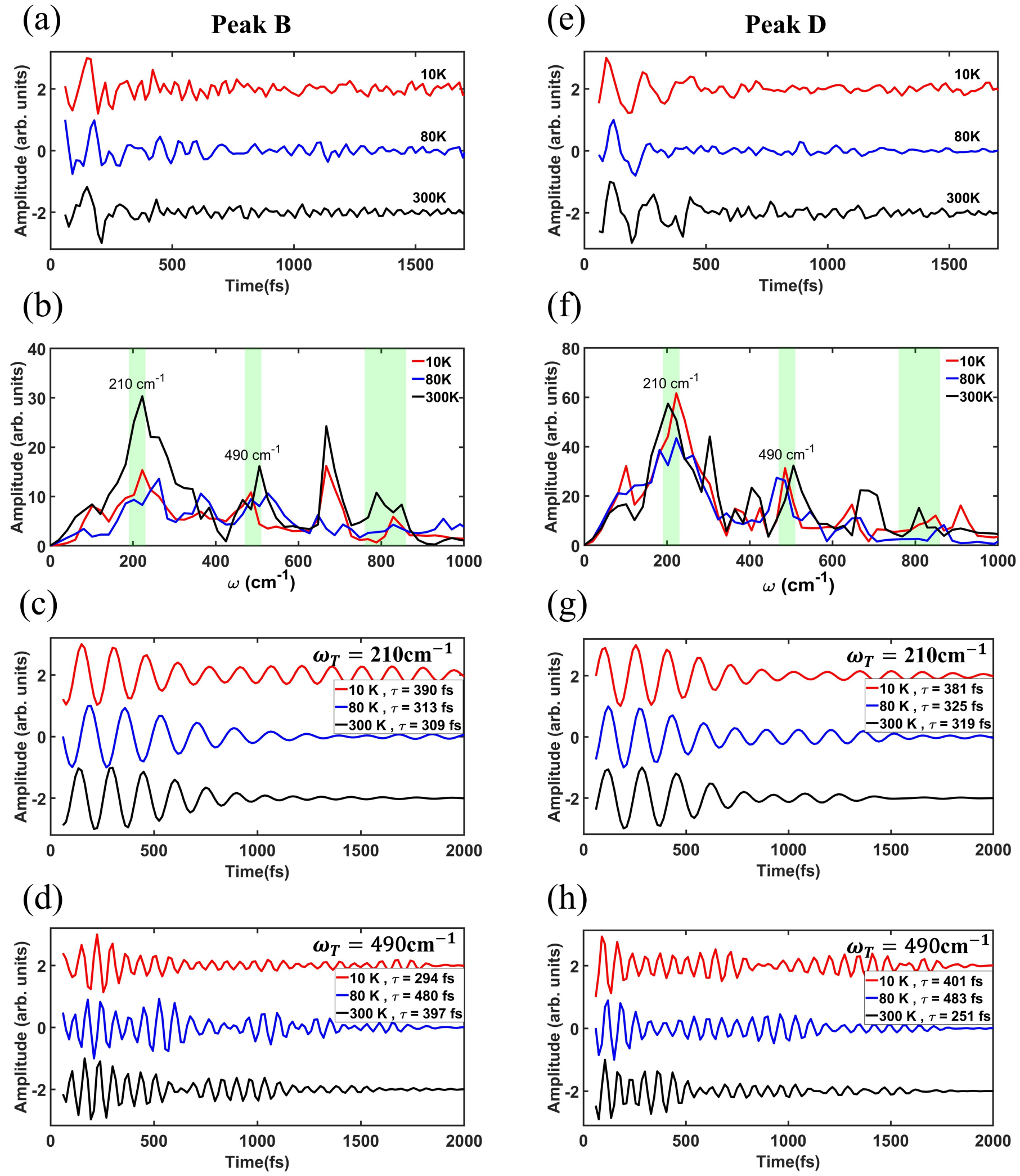

FIG. S6. (a) Residuals of peak B at different temperatures. (b) Corresponding Fourier spectra and selected vibrational bands. The vibrational coherence lifetimes of the 210 and 490 cm^{-1} bands are shown in (c) and (d), respectively. The corresponding residuals, Fourier spectra, and resolved lifetimes for peak D are shown in (e)–(h).

S8. WAVELET ANALYSIS

In this section, we summarize the basic principles of the wavelet transform, as described in Refs. (57, 58). The transform starts from a zero-mean, temporally localized oscillatory function $\boldsymbol{\psi}$, termed the "mother" wavelet, which decomposes a one- or multidimensional real-valued signal into different frequency bands. Translating this function in time by t and stretching it by the scaling factor ω^{-1} gives the wavelet atom

$$\psi_{t,\omega}(t') = \sqrt{\omega}\psi\left([t' - t]\omega\right). \tag{S4}$$

This function provides the basis for the transformation. The two most common forms are the discrete wavelet transform and the continuous wavelet transform (CWT) (56–58). The discrete transform decomposes a signal into several frequency bands and is frequently used for data and image compression. The CWT, which was used in this work, expands a temporal signal $f(t)$ through its inner product with a wavelet atom,

$$CWT_f(t,\omega) = \int_{-\infty}^{+\infty} dt' f(t')\sqrt{\omega}\psi^*\left([t' - t]\omega\right). \tag{S5}$$

The parameter t specifies the temporal center of the wavelet atom, whereas ω^{-1} controls its width relative to the mother wavelet. This nonlinear integral transform provides high temporal resolution for high-frequency components

and high frequency resolution for slowly varying components. It projects the signal onto basis functions with a variable center frequency and a frequency range determined by the scaling factor.

S9. TRANSIENT-GRATING (TG) SPECTRA AT DIFFERENT TEMPERATURES

In this section, we present the TG spectra of the APC trimer measured at 10, 80, and 300 K and analyze the coherent dynamics using time-resolved traces and wavelet analysis. The corresponding TG spectra are shown in Fig. S7(a), Fig. S8(a), and Fig. S9(a).

At 10 K, Fig. S7(b, e, h, k) shows the residuals and fits at selected probe wavelengths. Signals at $\lambda = 589$ and 671 nm correspond to excited-state absorption (ESA), whereas those at $\lambda = 611$ and 652 nm correspond to ground-state bleach (GSB). We Fourier-transformed the residuals to obtain the mode maps in Fig. S7(c, f, i, l) and applied the CWT to obtain the time-frequency maps in Fig. S7(d, g, j, m). Pronounced modes at 205, 456, and 821 cm^{-1} are evident at $\lambda = 589$ nm. At $\lambda = 611$ nm, modes at 273, 505, 662, and 867 cm^{-1} were observed. The maps at $\lambda = 652$ and 671 nm show modes at 273, 505, and 844 cm^{-1}.

At 80 K, Fig. S8(b, e, h, k) shows the residuals and fits at $\lambda = 593, 611, 652,$ and 671 nm. The corresponding mode maps and time-frequency maps are shown in Fig. S8(c, f, i, l) and Fig. S8(d, g, j, m), respectively. At 300 K, the analogous analysis was performed at $\lambda = 589$, 615, 662, and 681 nm (Fig. S9). At both temperatures, the lowest- and highest-wavelength channels correspond to ESA, and the two intermediate channels correspond to GSB.

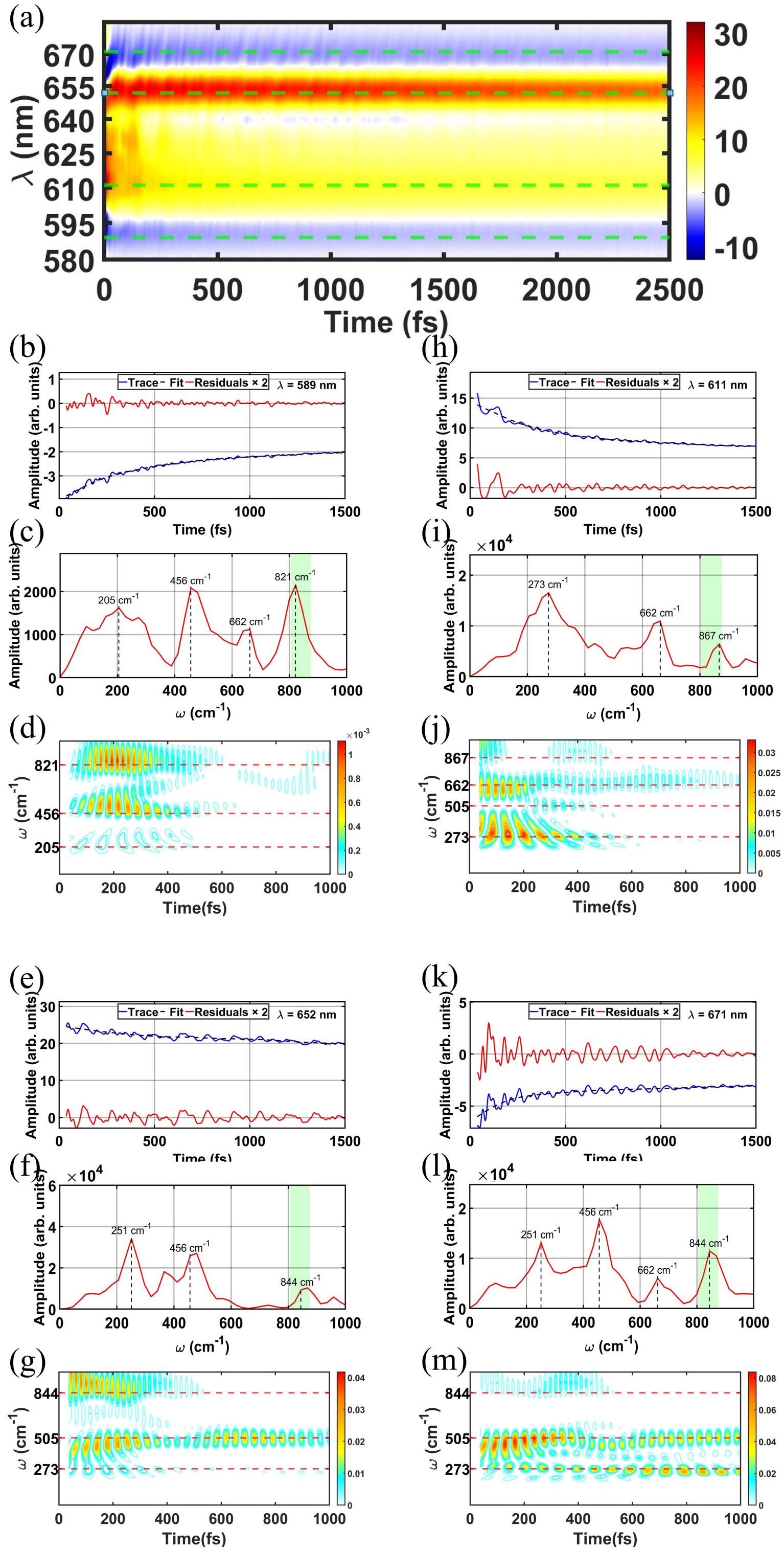

FIG. S7. Results at 10 K. (a) TG spectrum of the allophycocyanin trimer. (b, e, h, k) residuals and fits at selected probe wavelengths (ESA at $\lambda = 589$ nm and 671 nm; GSB at $\lambda = 611$ nm and 652 nm). (c, f, i, l) Mode maps from Fourier transforms of the residuals. (d, g, j, m) Continuous-wavelet time-frequency maps.

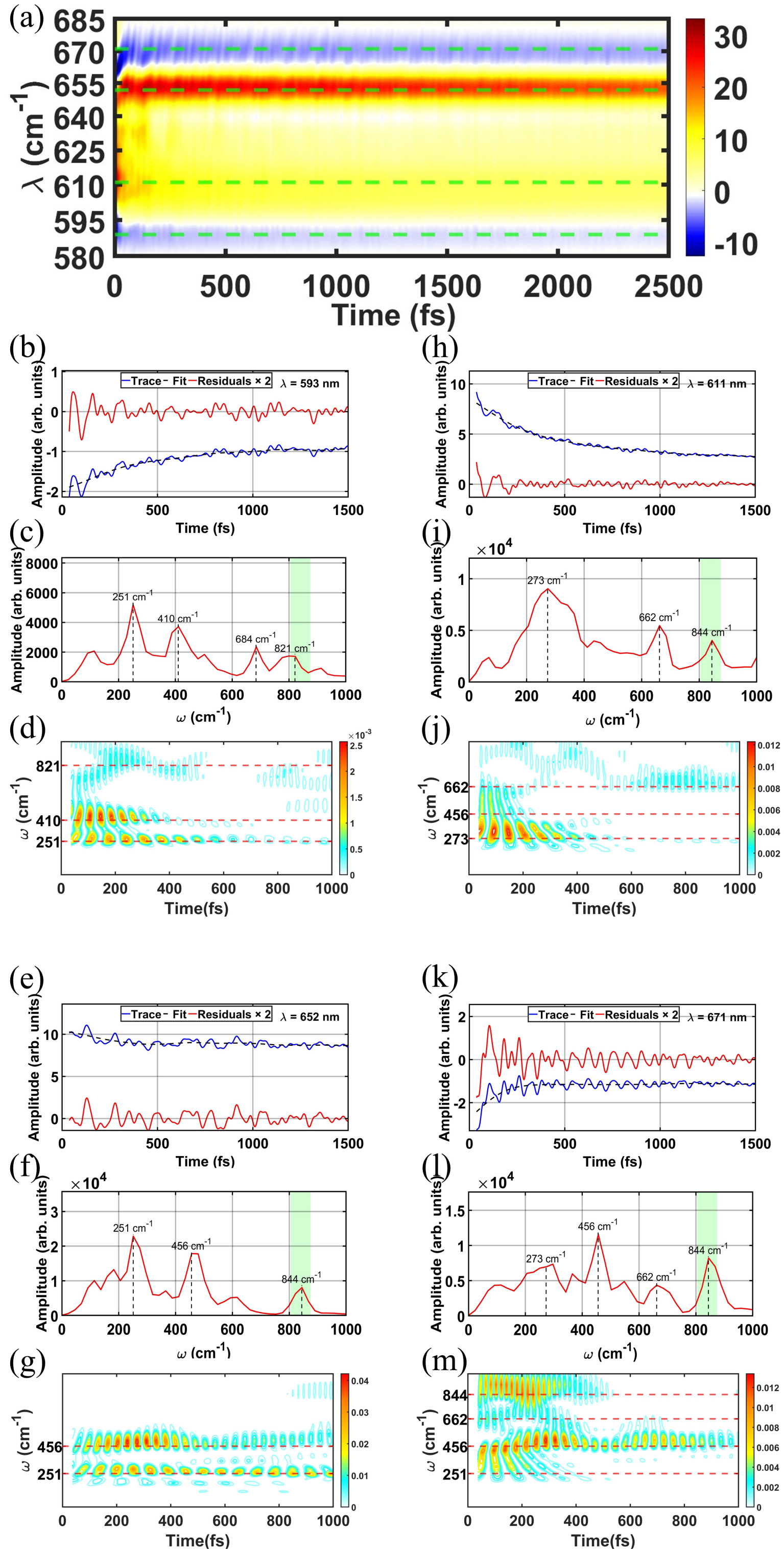

FIG. S8. Results at 80 K. (a) TG spectrum of the allophycocyanin trimer. (b, e, h, k) Residuals and fits at selected probe wavelengths (ESA at $\lambda = 593$ nm and 671 nm; GSB at $\lambda = 611$ nm and 652 nm). (c, f, i, l) Mode maps from Fourier transforms of the residuals. (d, g, j, m) Continuous-wavelet time-frequency maps.

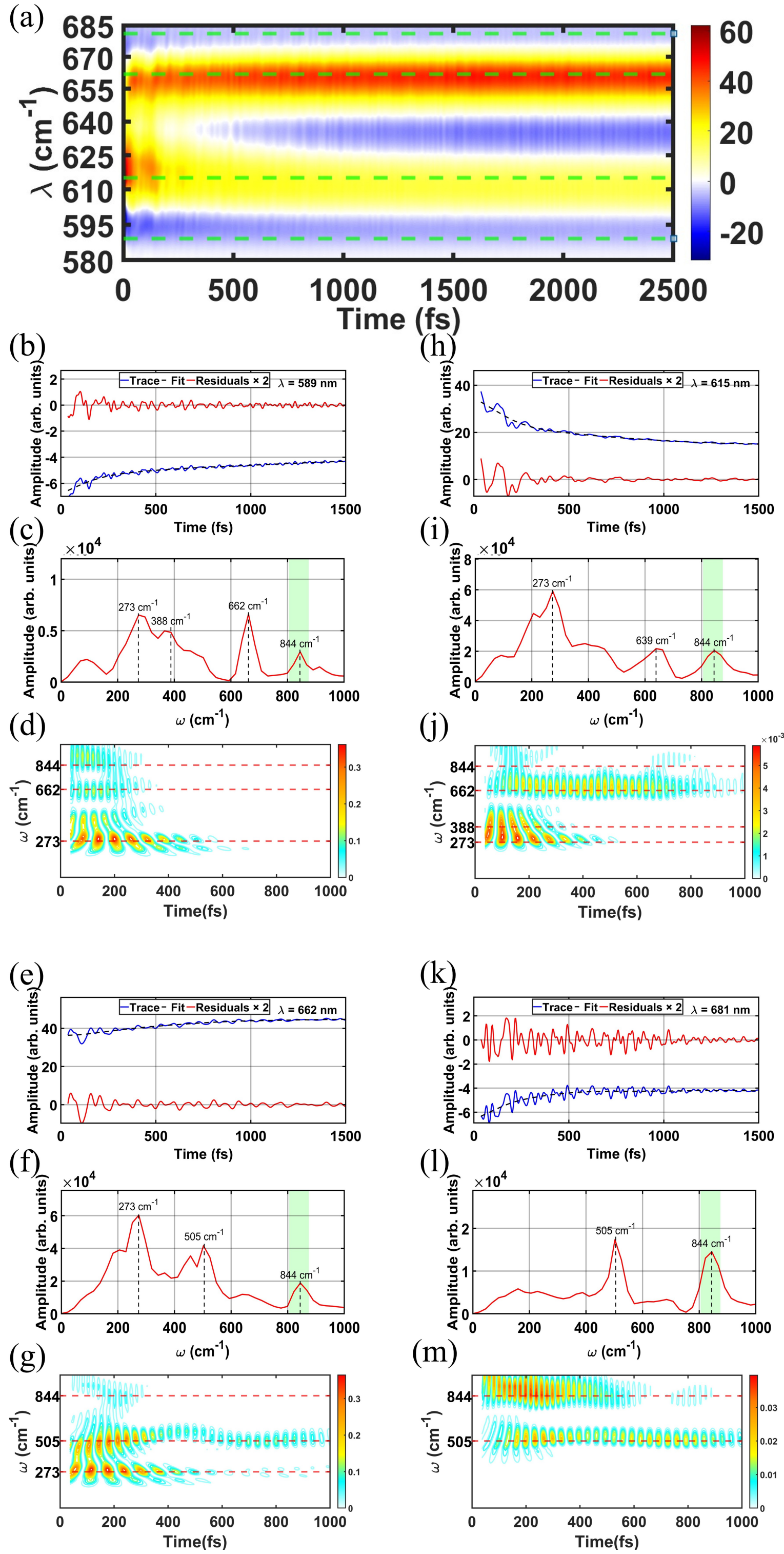

FIG. S9. Results at 300 K. (a) TG spectrum of the allophycocyanin trimer. (b, e, h, k) residuals and fits at selected probe wavelengths (ESA at $\lambda = 589$ nm and 681 nm; GSB at $\lambda = 615$ nm and 662 nm). (c, f, i, l) Mode maps from Fourier transforms of the residuals. (d, g, j, m) Continuous-wavelet time-frequency maps.

S10. ELECTRONIC AND VIBRATIONAL COHERENCE OF THE MODE AT 844 CM^{-1}

In this section, we describe the analysis of the 844 cm^{-1} mode. In the ESA region of the TG measurement (λ = 671 nm or λ = 681 nm), we observed a long-lived coherence at ω_T = 844 cm^{-1}; this frequency corresponds to a mixed electronic-vibrational coherence. We determined the lifetimes by fitting the oscillatory dynamics with an exponentially damped harmonic function. Representative results at 10 K, 80 K, and 300 K are shown in Fig. S10(a)–(c).

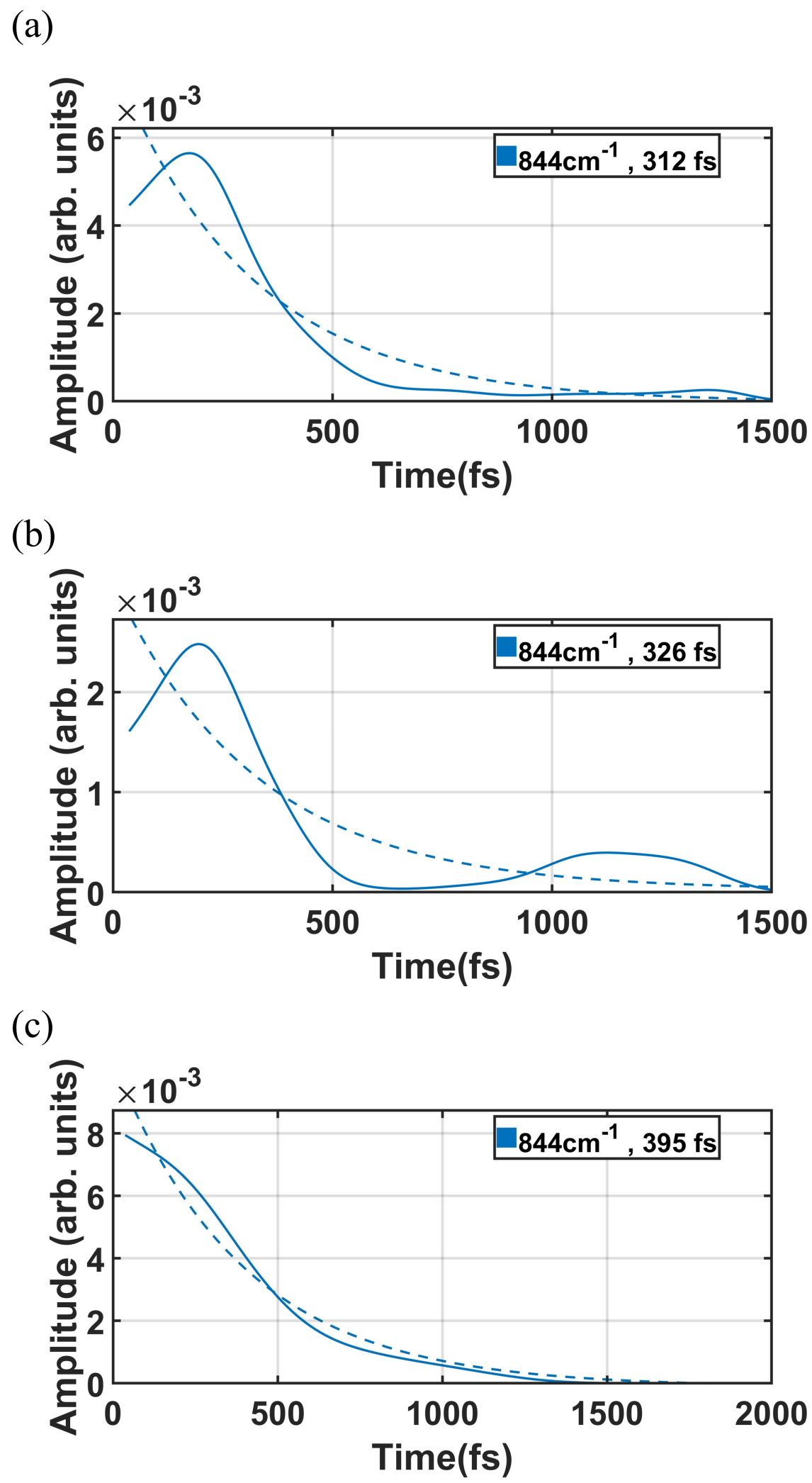

FIG. S10. (a) Coherence lifetime at ω_T = 844 cm^{-1} extracted via wavelet analysis from the ESA region of the TG trace (λ = 671 nm) at 10 K. (b) Coherence lifetime at ω_T = 844 cm^{-1} extracted via wavelet analysis from the ESA region of the TG trace (λ = 671 nm) at 80 K. (c) Coherence lifetime at ω_T = 844 cm^{-1} extracted via wavelet analysis from the ESA region of the TG trace (λ = 681 nm) at 300 K.

S11. AB INITIO CALCULATIONS OF THE APC TRIMER COMPLEX

All *ab initio* calculations were performed using Gaussian 16. The ground-state geometries of the α and β pigments were optimized at the CAM-B3LYP/def2-SVP level of theory. Subsequent vibrational frequency analysis was conducted at the same level to verify structural stability and characterize the key vibrational modes involved in the excitations.

Based on the optimized ground-state structures, time-dependent density functional theory (TD-DFT) calculations were performed using the same functional and basis set to obtain the first excited-state energies and transition dipole

moments of the two pigments (μ_α and μ_β). These transition dipole moments were used as input for the intermolecular excitonic coupling calculations. For the closest α/β pigment pair, with an intermolecular distance (r) of around 20 Å, the coupling strength was evaluated using the dipole–dipole interaction model (Eq. S6)

$$V = \frac{1}{4\pi\varepsilon_0\varepsilon_r r^3} \cdot [\mu_\alpha \cdot \mu_\beta - 3(\mu_\alpha \cdot \hat{r})(\mu_\beta \cdot \hat{r})] \,, \tag{S6}$$

yielding a value of ∼–157 cm^{-1}. Here, $\varepsilon_0 = 8.854 \times 10^{-12}$ F/m is the vacuum permittivity, $\varepsilon_r = 1$ corresponds to the relative permittivity of a vacuum environment, and $\hat{r}$ is the unit vector along the line connecting the centers of mass of the two pigments. For comparison, we also evaluated the excitonic coupling for the more distant α–β pigment pair in the trimer geometry, whose separation is approximately 50 Å. This pair corresponds to the α–β pigment pair in an APC monomer. Using the same dipole–dipole interaction model and TD-DFT transition dipole moments, the coupling strength was estimated to be only 9.64 cm^{-1}. This value is much smaller than the coupling of the closest inter-monomer pigment pair in the trimer (∼–157 cm^{-1}), indicating that the intra-monomer α–β excitonic coupling is very weak and can be neglected in the monomer model. Huang–Rhys factors were also calculated for the characteristic vibrational modes at 250 and 800 cm^{-1}. These factors were derived from the Franck–Condon displacements between the ground and excited states using vibrational-mode displacements and reduced masses extracted from the Gaussian 16 output files. The calculated vibrational motions of the α- and β-related pigments are shown in Fig. S11.

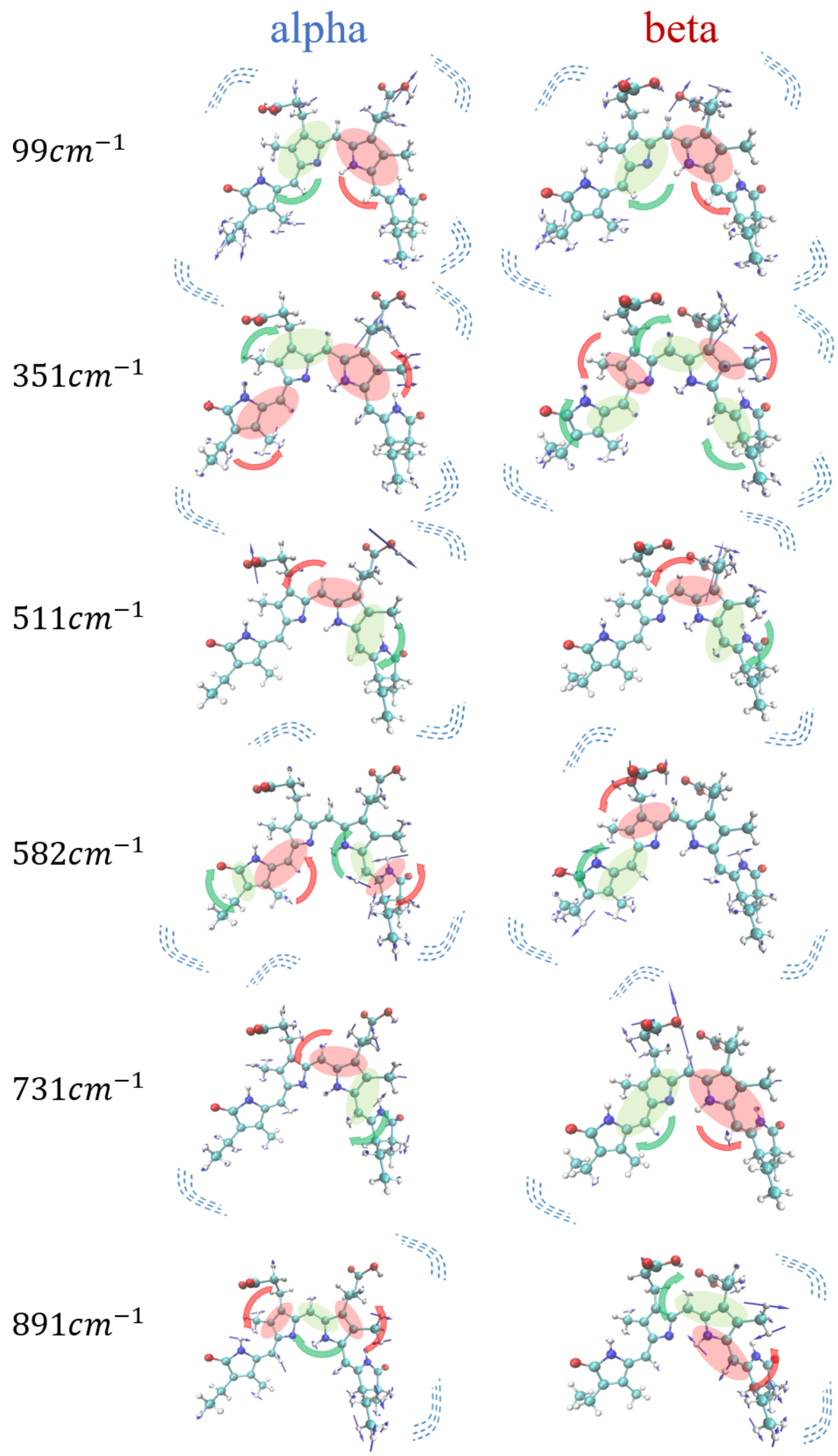

FIG. S11. Additional calculated vibrational motions of the α- and β-related pigments.

S12. HIERARCHICAL EQUATIONS OF MOTION

In this section, we summarize the Padé-HEOM formulation used for the dynamical simulations. Hamiltonian matrix elements, bath relaxation rates, Padé decay constants, and spectral-density parameters are reported in cm^{-1}. The thermal factor is written as

$$b_T = \frac{hc}{k_{\mathrm{B}}T}, \tag{S7}$$

so that $b_T\omega$ is dimensionless when ω is given in cm^{-1}.

For a system operator V_q coupled linearly to an independent harmonic bath q, the bath influence is determined by

the equilibrium bath correlation function

$$C_q(t) = \frac{1}{\pi}\int_0^\infty d\omega\, J_q(\omega)\left[\coth\left(\frac{b_T\omega}{2}\right)\cos(\omega t) - i\sin(\omega t)\right]. \tag{S8}$$

The key step in HEOM is to represent Eq. (S8) as a finite sum of exponential functions. For bath channel q, we used

$$C_q(t) = \sum_{k=0}^{K} c_{qk}\exp(-\nu_{qk}t) + 2\Delta_q\delta(t), \tag{S9}$$

where $k = 0$ denotes the Drude pole, $k \geq 1$ denotes the Padé poles, and K is the Padé expansion order used in the hierarchy. The residual term Δ_q accounts for the finite-order truncation of the real part of the bath correlation function and is included as a Markovian terminator.

For a Drude–Lorentz spectral density,

$$J_q(\omega) = \frac{2\lambda_q\gamma_q\omega}{\omega^2+\gamma_q^2}, \tag{S10}$$

the Drude pole is

$$\nu_{q0} = \gamma_q, \qquad c_{q0} = \lambda_q\gamma_q\left[\cot\left(\frac{b_T\gamma_q}{2}\right) - i\right]. \tag{S11}$$

The remaining Bose-function contribution was evaluated using the $[K/K]$ Padé decomposition,

$$\coth\left(\frac{x}{2}\right) \simeq \frac{2}{x} + 4x\sum_{k=1}^{K}\frac{\kappa_k}{x^2+\epsilon_k^2}, \qquad x = b_T\omega. \tag{S12}$$

Thus, the non-Drude decay constants are

$$\nu_{qk} = \frac{\epsilon_k}{b_T}, \qquad k = 1,\dots,K, \tag{S13}$$

and the corresponding coefficients are obtained from the residues of $J_q(z)\coth(b_Tz/2)$ at the Padé poles $z = i\nu_{qk}$,

$$c_{qk} = \frac{4\lambda_q\gamma_q}{b_T}\frac{\kappa_k\nu_{qk}}{\nu_{qk}^2-\gamma_q^2}, \qquad k = 1,\dots,K. \tag{S14}$$

The Padé constants ϵ_k and κ_k are temperature independent for a fixed expansion order K; temperature enters through b_T and therefore changes the Padé decay constants ν_{qk}.

The finite Padé expansion does not exactly reproduce the short-time real part of $C_q(t)$. The remaining contribution was included through the terminator coefficient Δ_q. In the same convention as the numerical implementation,

$$\Delta_q = \frac{2\lambda_q}{b_T\gamma_q}\left[1 - \frac{x_q}{\tan x_q} - \sum_{k=1}^{K}\frac{2\gamma_q^2\kappa_k}{\nu_{qk}^2-\gamma_q^2}\right], \qquad x_q = \frac{b_T\gamma_q}{2}. \tag{S15}$$

This expression for Δ_q follows the Padé normalization in Eq. (S12) and the Drude spectral-density convention in Eq. (S10). It gives the double-commutator contribution in Eq. (S16).

The system Hamiltonian H_S listed in the model sections below is the bare vibronic system Hamiltonian used in the propagation and does not already include the bath counter potential. Therefore, following the implementation, the counter term associated with the bilinear system–bath coupling was retained explicitly in the HEOM. With the present Drude convention, this contribution is equivalent to adding $\sum_q \lambda_q V_q^2$ to the coherent Liouvillian and appears as the commutator term in Eq. (S16). Since the counter term is not absorbed into H_S elsewhere, this does not double count the bath-induced renormalization.

With the exponential form in Eq. (S9), the auxiliary density operators are indexed by $\mathbf{n} = \{n_{qk}\}$, where n_{qk} is a nonnegative integer. The physical reduced density matrix is $\rho_{\mathbf{0}}(t)$. The HEOM used in the simulations can be written

as

$$
\begin{aligned}
\frac{d}{dt}\rho_{\mathbf{n}}(t) = &-\left(i\mathcal{L}_S + \sum_q \sum_{k=0}^{K} n_{qk}\nu_{qk}\right)\rho_{\mathbf{n}}(t) - i\sum_q \left[V_q, \sum_{k=0}^{K}\rho_{\mathbf{n}+\mathbf{e}_{qk}}(t)\right] \\
&- i\sum_q \sum_{k=0}^{K} n_{qk}\left[c_{qk}V_q\rho_{\mathbf{n}-\mathbf{e}_{qk}}(t) - c_{qk}^{*}\rho_{\mathbf{n}-\mathbf{e}_{qk}}(t)V_q\right] - i\sum_q \lambda_q\left[V_q^2, \rho_{\mathbf{n}}(t)\right] \\
&- \sum_q \Delta_q\left[V_q, \left[V_q, \rho_{\mathbf{n}}(t)\right]\right],
\end{aligned}
\tag{S16}
$$

where $\mathcal{L}_S\rho = [H_S, \rho]$, V_q is the system operator coupled to bath q, and $\mathbf{e}_{qk}$ increases the n_{qk} component by one. The terms proportional to $i\mathcal{L}_S$ and $n_{qk}\nu_{qk}$ describe coherent system evolution and exponential damping of each auxiliary density operator. The term involving $\rho_{\mathbf{n}+\mathbf{e}_{qk}}$ couples the current auxiliary density operator to the next tier of the hierarchy, whereas the term involving $\rho_{\mathbf{n}-\mathbf{e}_{qk}}$ couples it to the previous tier through the Padé residues. The commutator with V_q^2 is the counter-term correction, and the double commutator proportional to Δ_q is the Markovian terminator for the residual correlation function.

The hierarchy was truncated by a total-tier cutoff,

$$
\sum_q \sum_{k=0}^{K} n_{qk} \le N_{\text{tier}},
\tag{S17}
$$

which retains the leading non-Markovian bath-memory contributions while keeping the calculation finite. In the temperature-dependent Drude–Lorentz model, each low- or high-frequency Drude component is treated as a separate bath channel q, so that the number of hierarchy indices increases with the number of bath components.

S13. MODELING AND REFINED PARAMETERS

In this section, we summarize the model Hamiltonian and the refined parameters used in the calculations. The Hamiltonian comprises four electronic states: the ground state g, the electronic state S_1 representing the β subunit, the electronic state S_2 representing the α subunit, and the double-excitation state S_{12}. For the calculations, we selected two key vibrational modes with frequencies of 250 and 800 cm^{-1}. For the mode with $\Omega_t = 250$ cm^{-1}, the vibronic coupling parameters obtained from the *ab initio* calculations are $\kappa_{S1} = 247.0$ cm^{-1} and $\kappa_{S2} = 80.0$ cm^{-1}. For the mode with $\Omega_t = 800$ cm^{-1}, the corresponding values are $\kappa_{S1} = 137.0$ cm^{-1} and $\kappa_{S2} = 40.0$ cm^{-1}. The site energies are $\epsilon_{S1} = 16060$ cm^{-1} and $\epsilon_{S2} = 15300$ cm^{-1}. In addition, we assume that the electronic system is linearly coupled to two independent thermal baths, leading to the system–bath interaction terms $H_{sb1} = (|S_1\rangle\langle S_1| + |S_{12}\rangle\langle S_{12}|)\sum_j c_j x_j$ and $H_{sb2} = (|S_2\rangle\langle S_2| + |S_{12}\rangle\langle S_{12}|)\sum_{j'} c_{j'} x_{j'}$. We employed a Drude–Lorentz (Lorentzian) spectral density with parameters $\lambda_{S1} = 200$ cm^{-1}, $\lambda_{S2} = 80$ cm^{-1}, $\gamma_{S1} = 100$ cm^{-1}, and $\gamma_{S2} = 100$ cm^{-1}, and included static disorder of approximately 20 cm^{-1}. For each selected vibrational mode, five vibrational levels were retained, and we confirmed that the wavepacket population does not significantly reach beyond this truncated vibrational manifold, indicating convergence with respect to the vibrational basis size. For the HEOM calculations, two independent baths were included, and the finite-temperature bath correlation function of each bath was represented using a Padé decomposition, with one auxiliary exponential term retained in addition to the Drude contribution. This corresponds to a maximum hierarchy tier of 7 in the present calculations. The propagation time step was set to 1 fs. We verified convergence with respect to both the hierarchy depth and the bath-correlation expansion: increasing the hierarchy tier further leads to numerically stable results, while the calculations are primarily sensitive to the finite-temperature representation of bath memory. In particular, at 80 K, numerical convergence is achieved only when the number of retained auxiliary exponential terms for each bath is increased from one to two.

S14. PEAKS TAKEN FROM THE CALCULATED 2DES

In this section, we present the calculated time-resolved traces for peaks C and D, complementing peaks A and B in Fig. 5 of the main text. The traces are shown as black squares in Fig. S12(a) and Fig. S12(b), with fits and residuals plotted as blue and red solid lines, respectively. The Fourier-transform spectra are shown in Fig. S12(c) and Fig. S12(d), and the corresponding wavelet analyses are shown in Fig. S12(e) and Fig. S12(f). Frequencies of 234 and 819 cm^{-1} were resolved.

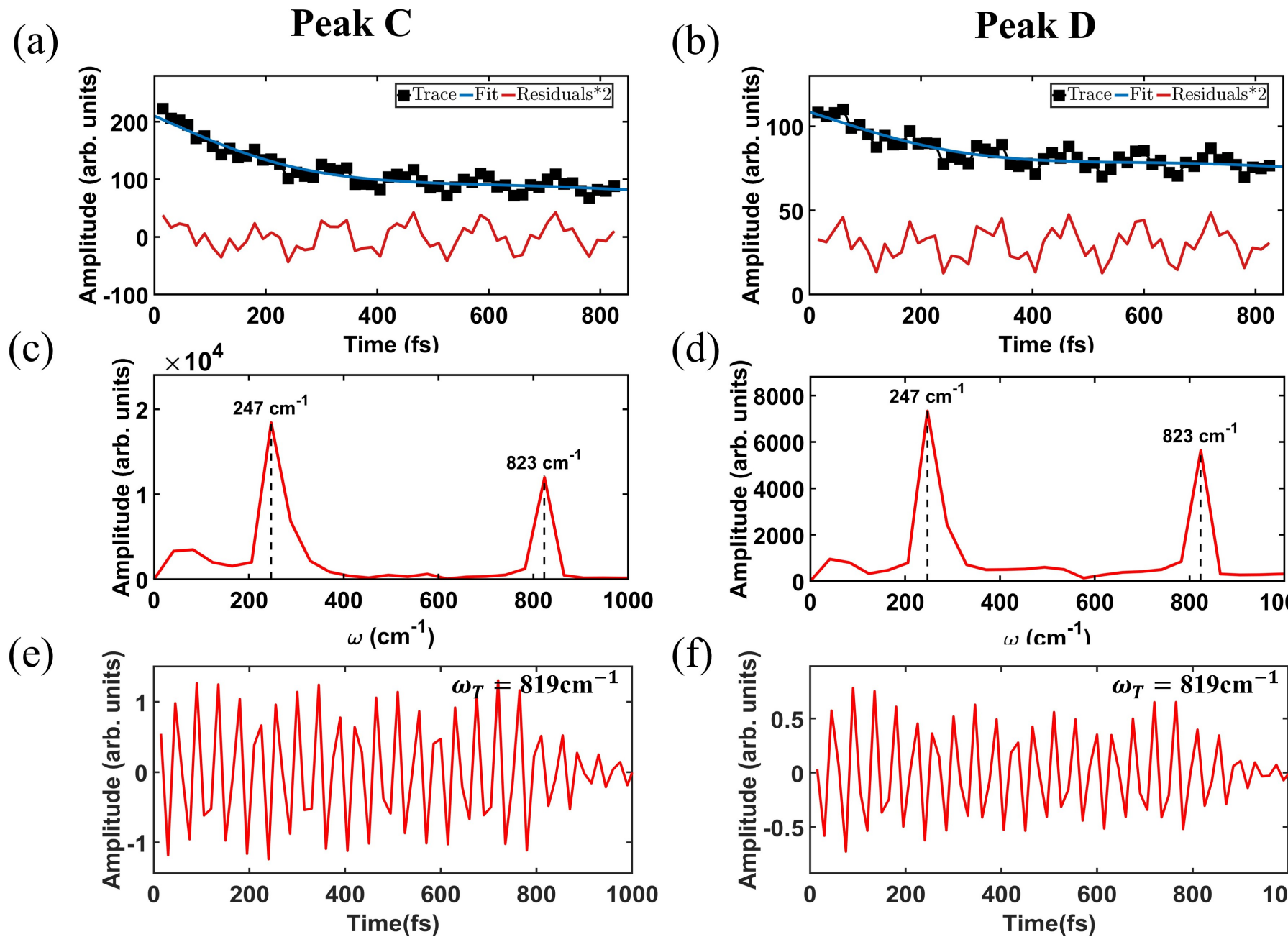

FIG. S12. Time-resolved traces of selected peaks (a) C and (b) D. The fits and residuals are shown as blue and red solid lines, respectively. (c, d) Fourier-transform spectra of the residuals. (e, f) Wavelet amplitudes of the 819 cm^{-1} mode.

S15. POPULATION DYNAMICS CALCULATED WITH DIFFERENT VIBRATIONAL FREQUENCIES

In this section, we calculated the population dynamics using the refined model and examined the electronic and vibrational coherences during population transfer (Fig. S13). We first reduced the system–bath coupling strengths to 0.5 times the refined values. The calculations showed oscillations with larger amplitudes and longer lifetimes. We did not observe selective enhancement when the vibrational frequency approached the 790 cm^{-1} excitonic energy gap. Instead, the oscillatory dynamics varied smoothly as the vibrational frequency was changed from 700 to 900 cm^{-1}.

We then calculated the oscillatory dynamics using the refined system–bath coupling strengths (Fig. S14). Relative to the weaker-coupling case, the coherence amplitudes and lifetimes were reduced. Increasing the system–bath coupling strengths to 1.5 times the refined values reduced them further (Fig. S15). Across the three coupling strengths, the oscillation amplitudes and lifetimes changed smoothly with the system–bath interaction. Thus, we believe that electronic coherence cannot be enhanced or prolonged by vibrational coherence, even in the resonant case.

We next calculated the population dynamics for different system–bath coupling strengths, λ, and plotted the diagonal elements of the time-resolved density matrix in Fig. S16. At 0.5λ, the fitted population-transfer timescales were 247 fs for the standard vibronic coupling and 935 fs for the weak-coupling case ($\Delta = 0.01$; Fig. S16(a)). At λ, the corresponding timescales were 147 and 325 fs (Fig. S16(b)). At 1.5λ, they were 117 and 201 fs, respectively (Fig. S16(c)). Thus, the difference between the two fitted timescales within Fig. S16(c) is approximately 84 fs, smaller than the corresponding differences at 0.5λ and λ. Across all three conditions, the standard vibronic coupling accelerated population transfer relative to the weak-coupling case, whereas the absolute transfer times also depended strongly on λ.

We also projected the wave-packet dynamics of the electronic states onto the vibrational reaction coordinates (Fig. S17). The resolved oscillation periods match the assigned vibrational modes in the model. The states $|e_1\rangle$ and $|e_2\rangle$ represent the electronic states of the α and β pigments, respectively. The coordinates Q_1 and Q_2 represent the 800 and 250 cm^{-1} modes, respectively.

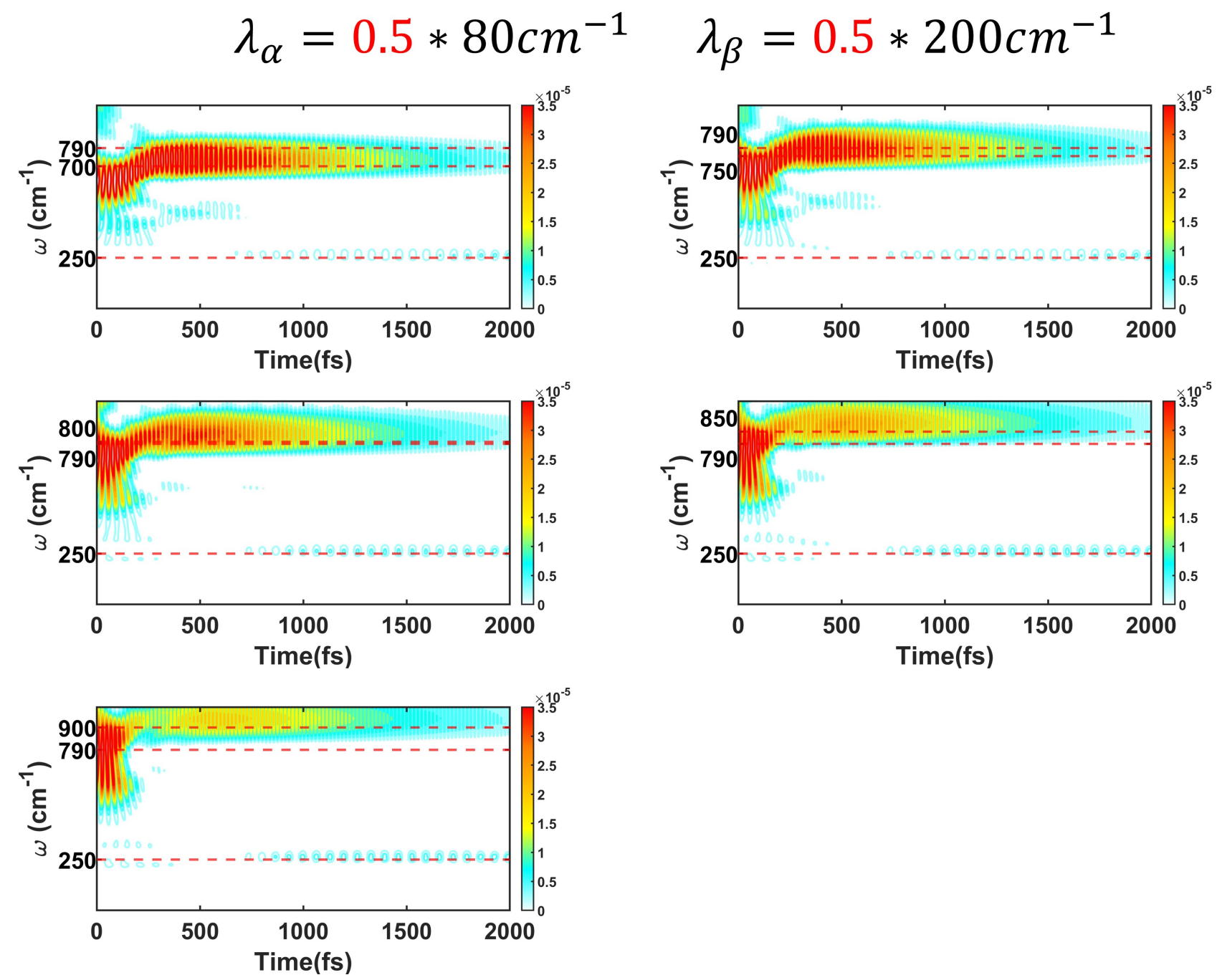

FIG. S13. Calculated population dynamics for system–bath coupling strengths set to 0.5 times the refined values. The weaker coupling produces larger coherence amplitudes and longer lifetimes. The electronic coherence is resolved at 790 cm^{-1}, and the vibrational frequency is varied from 700 to 900 cm^{-1}.

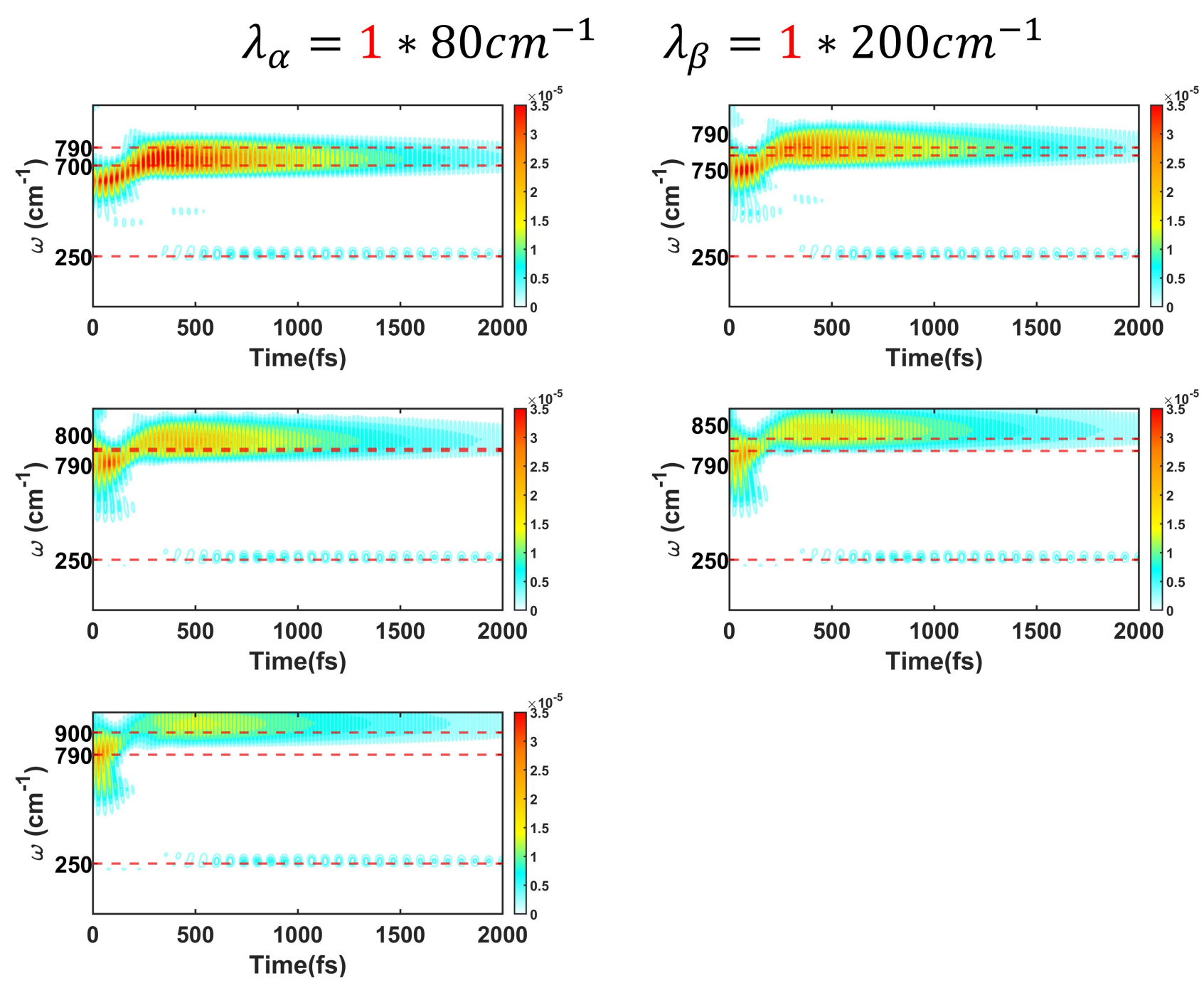

FIG. S14. Calculated population dynamics and resolved electronic and vibrational coherences obtained using the refined model parameters. The coherence persists for approximately 800 fs.

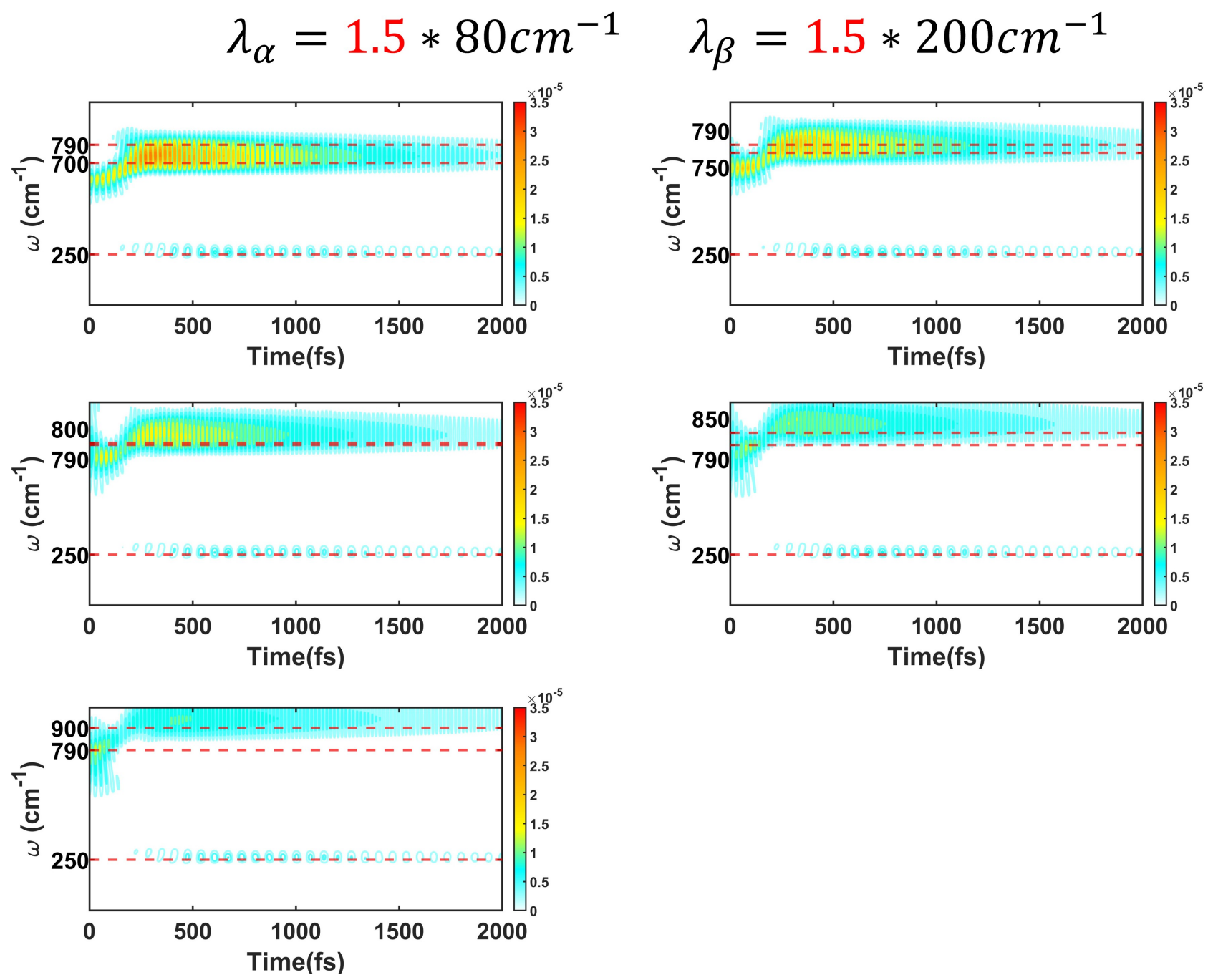

FIG. S15. Calculated population dynamics for system–bath coupling strengths set to 1.5 times the refined values. The coherence amplitude and lifetime are reduced, and the coherence persists for approximately 400 fs.

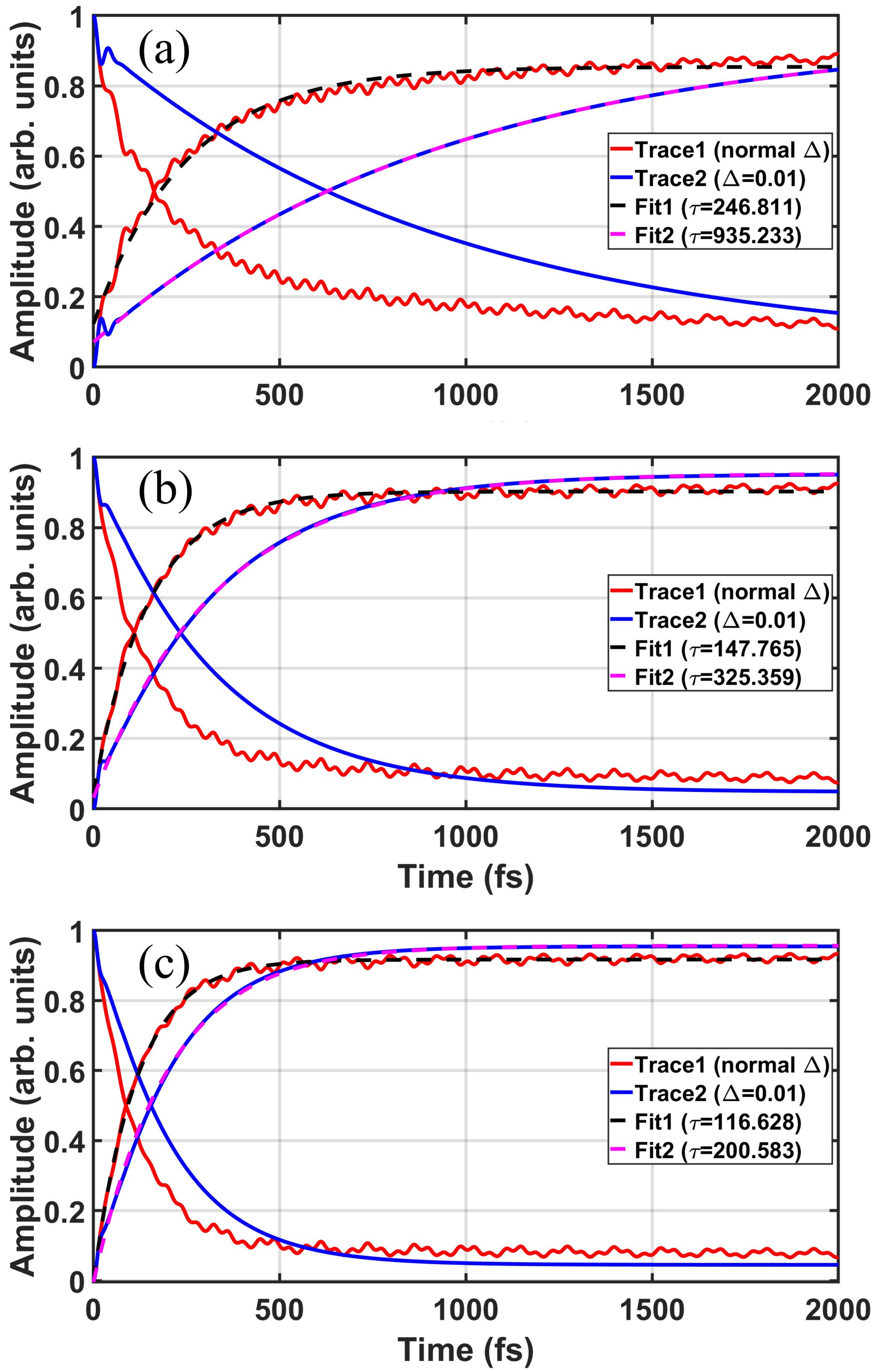

FIG. S16. Population transfer from the upper to the lower excited state at different system–bath coupling strengths, λ. Transfer timescales were obtained by exponential fitting and compared with those for weak vibronic coupling, $\Delta = 0.01$.

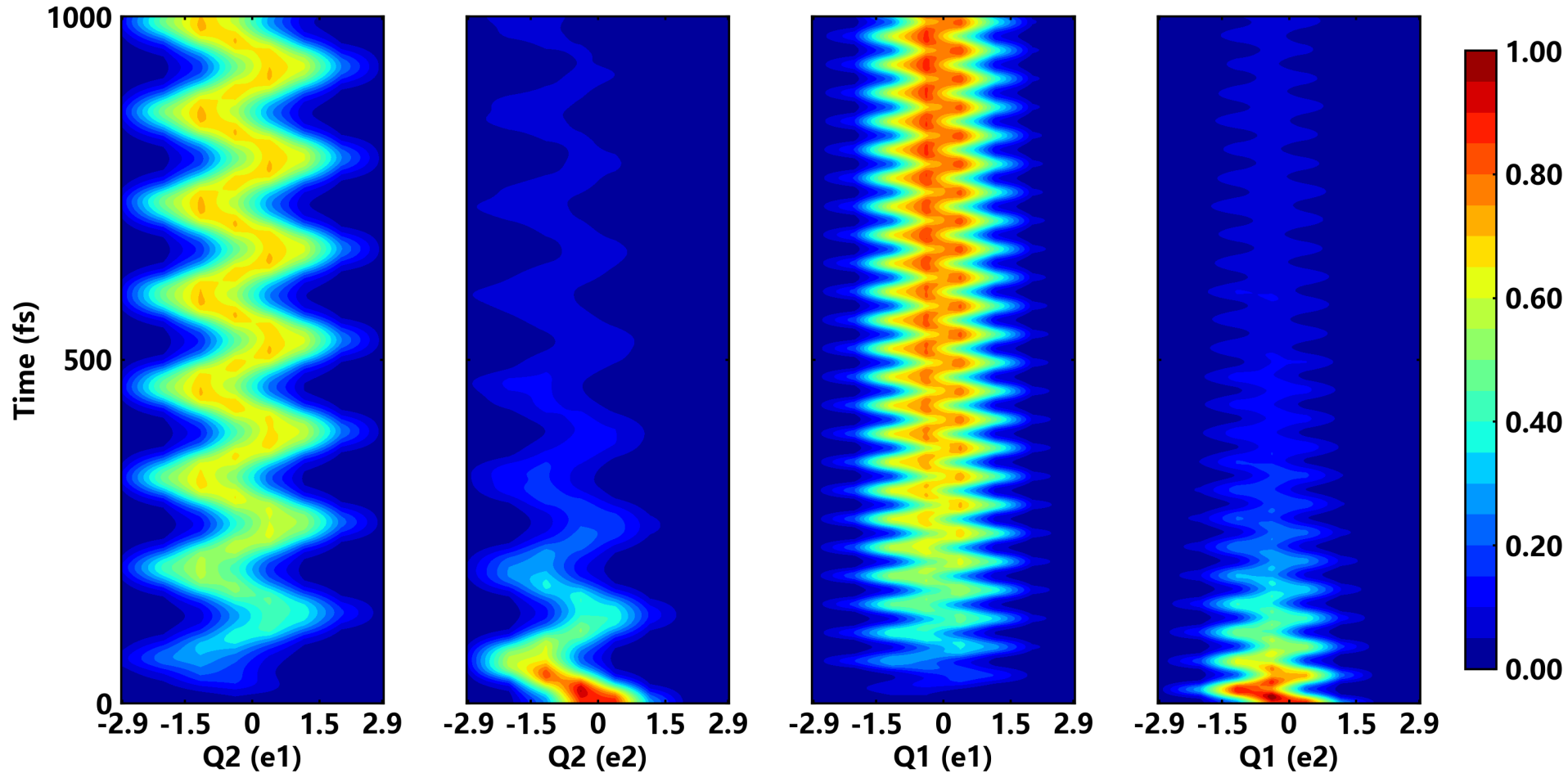

FIG. S17. Wave-packet dynamics from the upper to the lower electronic excited state, projected onto the vibrational modes with frequencies Ω_1 and Ω_2.

S16. 2DES CALCULATED AT 80 K

In this section, we calculated the 2DES of the APC trimer at 80 K. The resulting spectra are shown in Fig. S18.

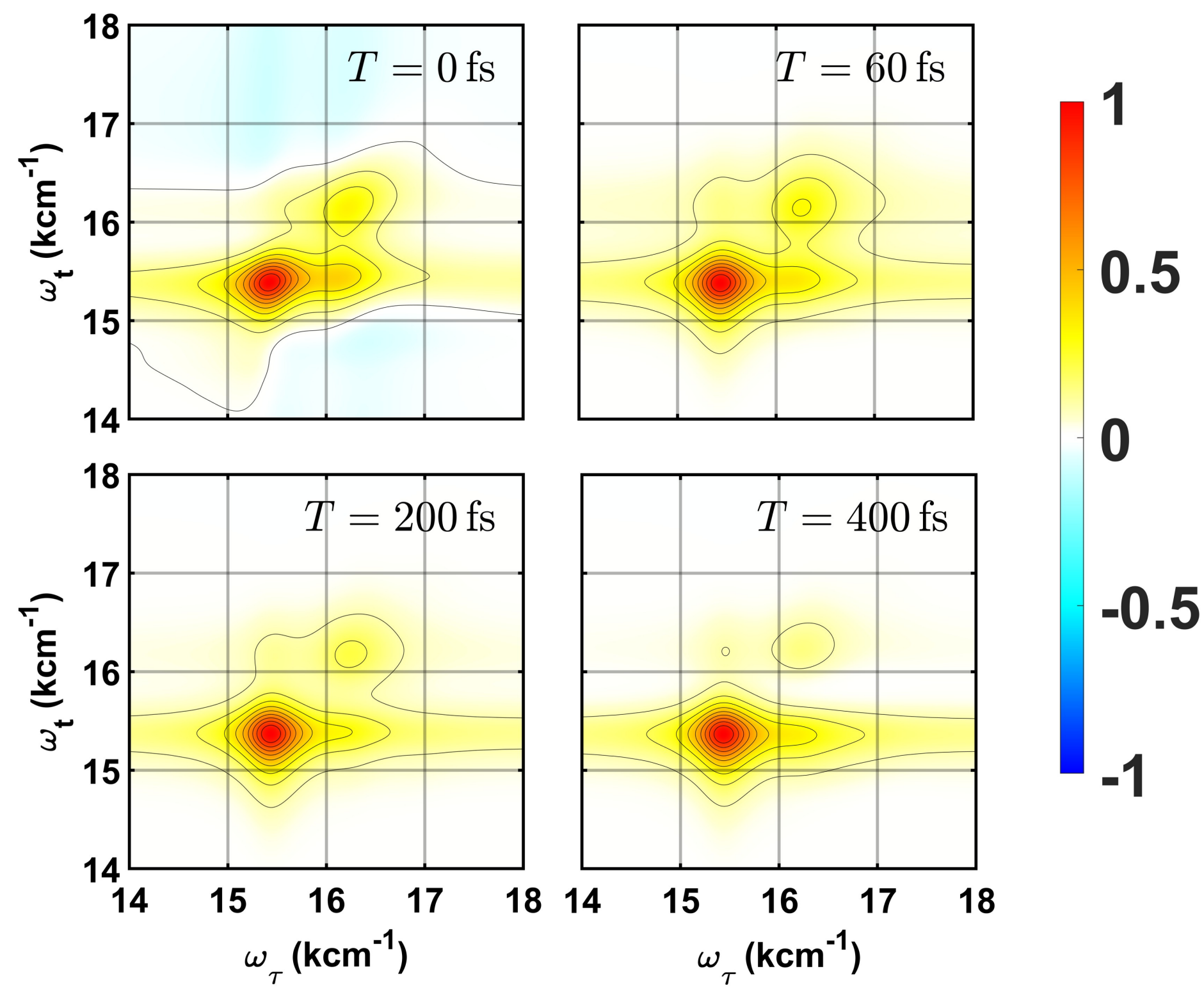

FIG. S18. Calculated 2DES of the APC trimer at 80 K at waiting times of 0, 60, 200, and 400 fs.

S17. COHERENT DYNAMICS IN MONOMERIC APC

To examine purely vibrational coherence, we prepared monomeric APC. Recombinant APC (rAPC) was obtained from the same supplier described in the Materials and Methods section of the main text. The rAPC monomer was prepared by disassembling the rAPC trimer in 8 M urea containing 20 mM phosphate and 2 mM EDTA. The linear absorption spectrum of the rAPC monomer was measured in a 3-mm-path-length cuvette at room temperature and is shown in Fig. S19.

A. Transient grating measurements

The transient absorption (TA) and TG spectra were collected by scanning the delay from −200 fs to 3 ps in 3-fs steps. At each delay, 200 spectra were averaged to improve the signal-to-noise ratio. Figure S20 summarizes the TG measurements on the APC monomer (APC-m). Figure S20(a) shows the spectrally resolved TG signal, with probe wavelengths of 612 and 639 nm indicated. Figures S20(b) and S20(c) show the corresponding TG traces, fits, and residuals. Figures S20(d) and S20(e) show the Fourier spectra of the residuals, and Fig. S20(f) and Fig. S20(g) show the corresponding wavelet time-frequency maps.

B. Two-dimensional electronic spectra of monomeric APC

For the 2DES measurements, the coherence-time scan, τ, was performed from −64 to 64 fs. The waiting time was scanned from 0 to 2 ps in 15-fs steps. The excitation-pulse energy was attenuated to 10 nJ at a repetition rate of 1

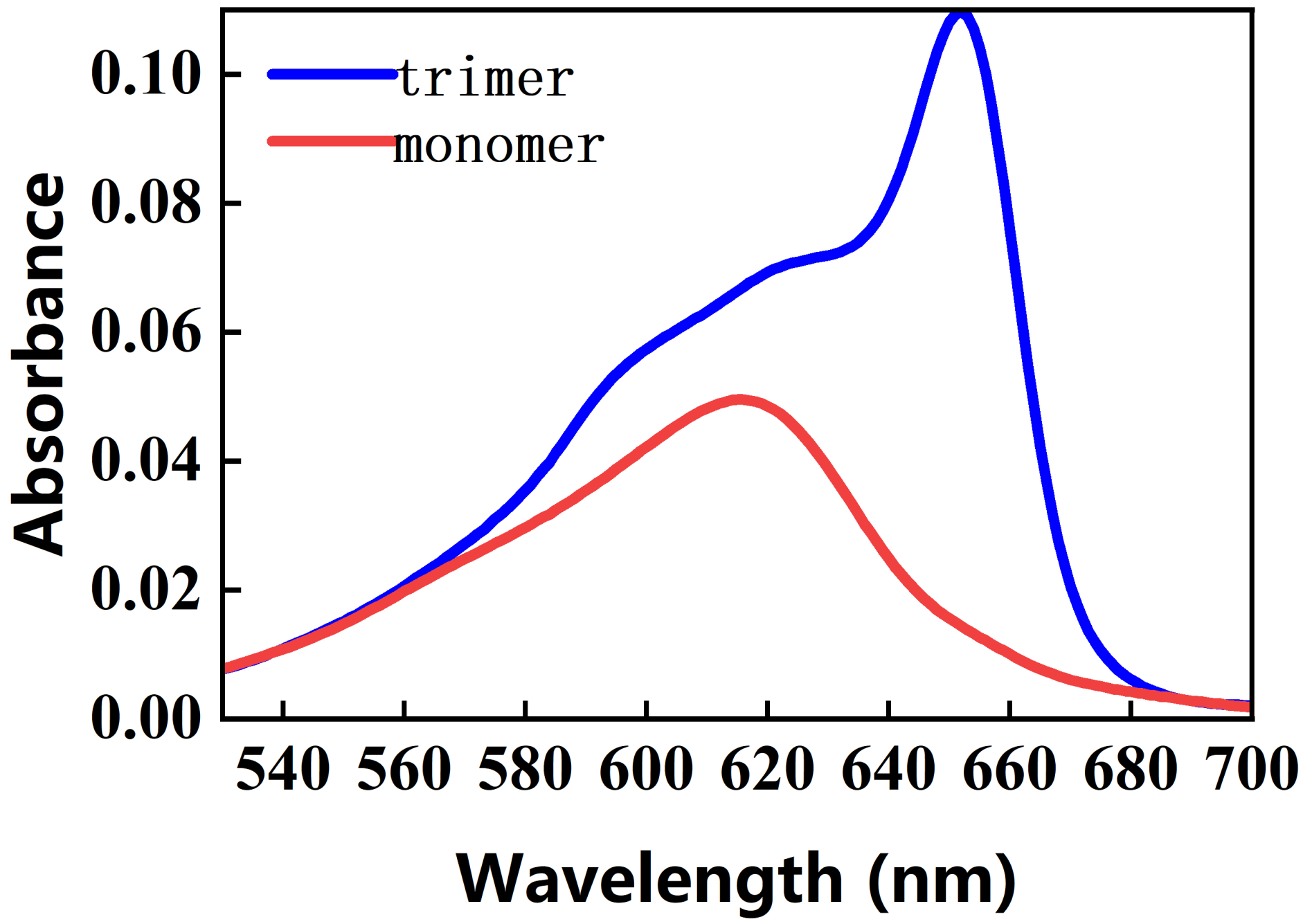

FIG. S19. Measured absorption spectrum of the APC monomer (red line). The absorption spectrum of the rAPC trimer (blue line) is shown for comparison.

kHz for all measurements. The room-temperature 2DES of the APC monomer are shown in Fig. S21. Figure S21(a) shows the real part of the APC-m 2DES. Figures S21(b) and S21(c) show the time traces, fits, and residuals at peaks A and B, respectively. Figures S21(d) and S21(e) show the corresponding Fourier spectra.

C. Theoretical calculations of 2DES of APC-m

For the monomeric APC model, the total Hamiltonian retains the usual form

$$H = H_S + H_B + H_{SB}, \tag{S18}$$

while the system Hamiltonian is simplified by neglecting the weak intra-monomer excitonic coupling between the α and β chromophores,

$$H_S = \sum_{i=1}^{n} \Big(|a_i\rangle (\epsilon_a + h_i) \langle a_i| + |b_i\rangle (\epsilon_b + h_i') \langle b_i| + |a_i b_i\rangle (\epsilon_a + \epsilon_b + h_i + h_i') \langle a_i b_i| \Big). \tag{S19}$$

Here, $|a\rangle$ and $|b\rangle$ denote the vibronic states of the monomeric APC chromophores. The electronic energies were set to $\epsilon_a = 15300$ cm^{-1} and $\epsilon_b = 16060$ cm^{-1}. No inter-pigment coupling term was included in the monomer model.

The site-local vibrational Hamiltonian is written as

$$h_i = \sum_{m}^{2} \frac{1}{2}\hbar\Omega_m \left(\alpha_{i,m}^{\dagger}\alpha_{i,m} + \frac{1}{2} \right) + \kappa_m Q_m, \tag{S20}$$

$$h_m^{'} = \sum_{m}^{2} \frac{1}{2}\hbar\Omega_m \left(\alpha_{i,m}^{\dagger}\alpha_{i,m} + \frac{1}{2} \right) + \kappa_m^{'} Q_m, \tag{S21}$$

where $\Omega_1 = 250$ cm^{-1} and $\Omega_2 = 800$ cm^{-1} are the dominant intramolecular mode and κ_m describes the vibronic coupling at the site.

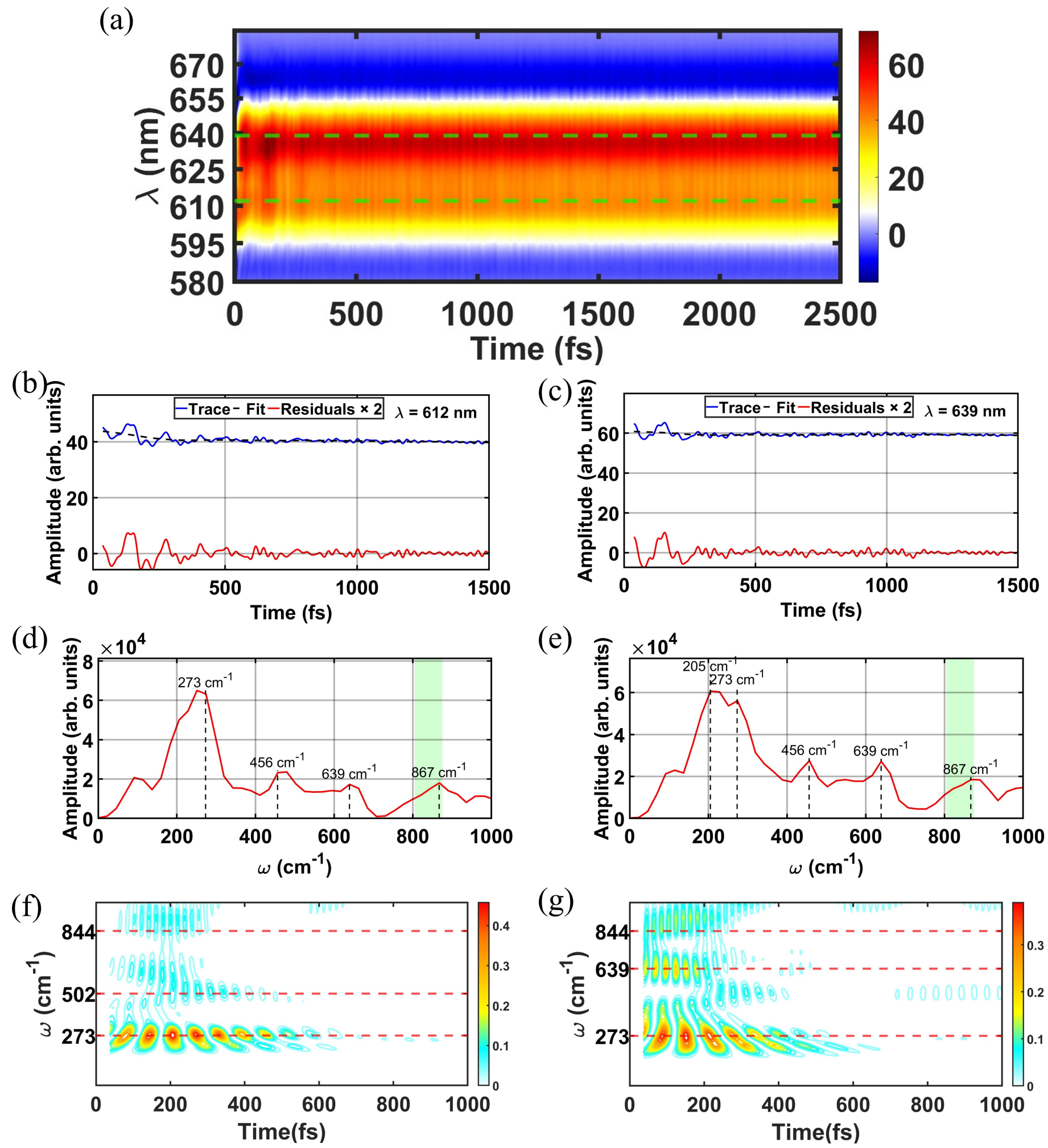

FIG. S20. Transient grating measurements on the APC monomer (APC-m). (a) Spectrally resolved TG signal with probe wavelengths at 612 nm and 639 nm indicated. (b) TG trace, fit, and residuals at 612 nm. (c) TG trace, fit, and residuals at 639 nm. (d) Mode spectrum obtained from the Fourier analysis of the residuals at 612 nm. (e) Mode spectrum obtained from the Fourier analysis of the residuals at 639 nm. (f) Wavelet time-frequency map for the residuals at 612 nm. (g) Wavelet time-frequency map for the residuals at 639 nm.

The bath and system–bath interaction follow the standard harmonic form,

$$H_B = \sum_i \left(\frac{p_i^2}{2m_i} + \tfrac{1}{2} m_i \omega_i^2 x_i^2 \right), \qquad H_{SB} = \sum_i K_i c_i x_i, \tag{S22}$$

with spectral density

$$J(\omega) = \pi \sum_i \frac{c_i^2}{2m_i\omega_i} \delta(\omega - \omega_i). \tag{S23}$$

The calculated 2DES of the APC monomer are shown in Fig. S22. Figure S22(a) shows the calculated real part of the APC-m 2DES. Figure S22(b) shows the simulated time trace, multiexponential fit, and residuals at peak A. Figure S22(c) shows the corresponding Fourier spectrum.

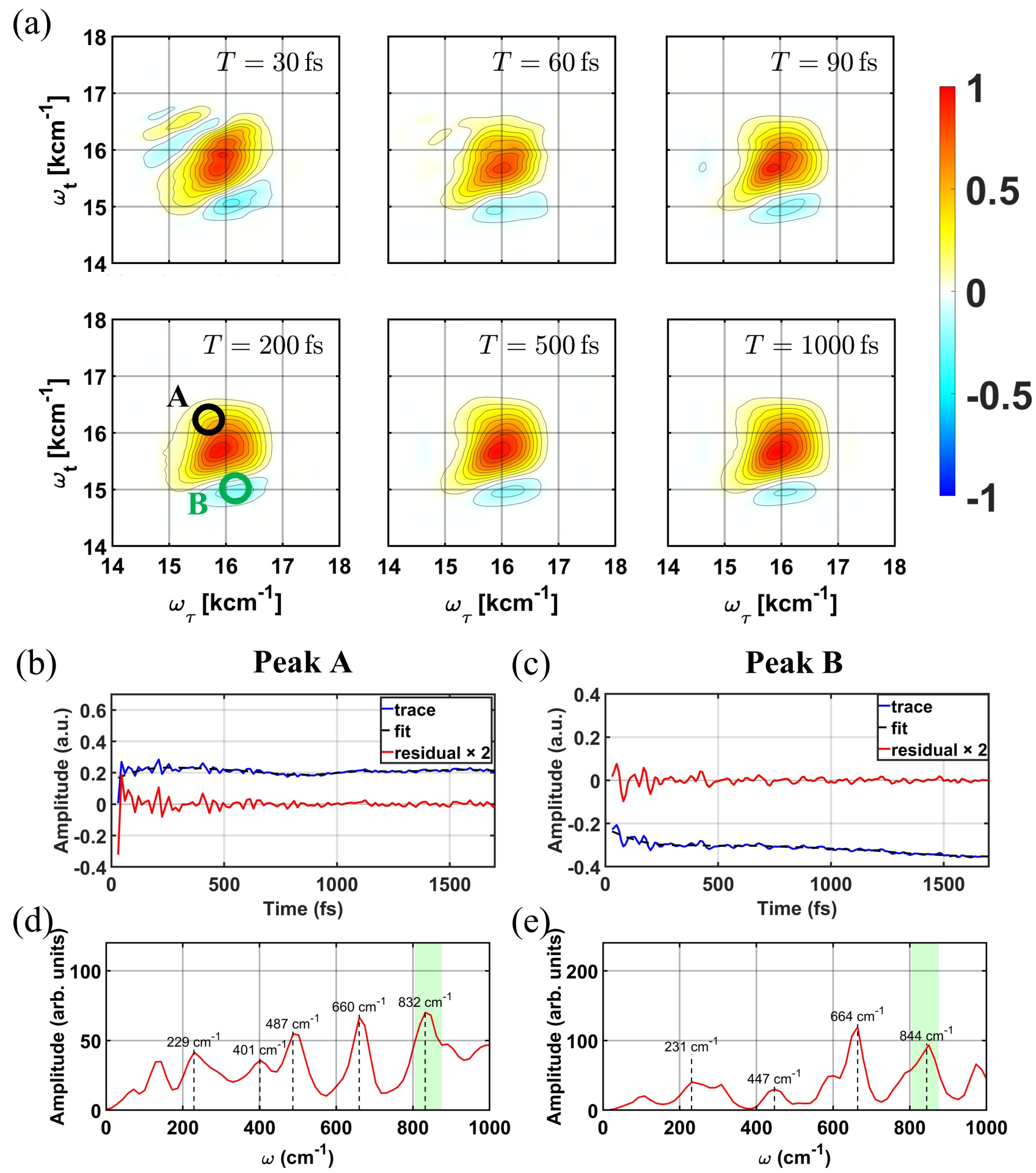

FIG. S21. Two-dimensional electronic spectra of the APC monomer. (a) Real part of the APC-m 2DES. (b) Time trace, fit, and residuals at peak A. (c) Time trace, fit, and residuals at peak B. (d) Mode spectrum obtained from the Fourier analysis of the residuals at peak A. (e) Mode spectrum obtained from the Fourier analysis of the residuals at peak B.

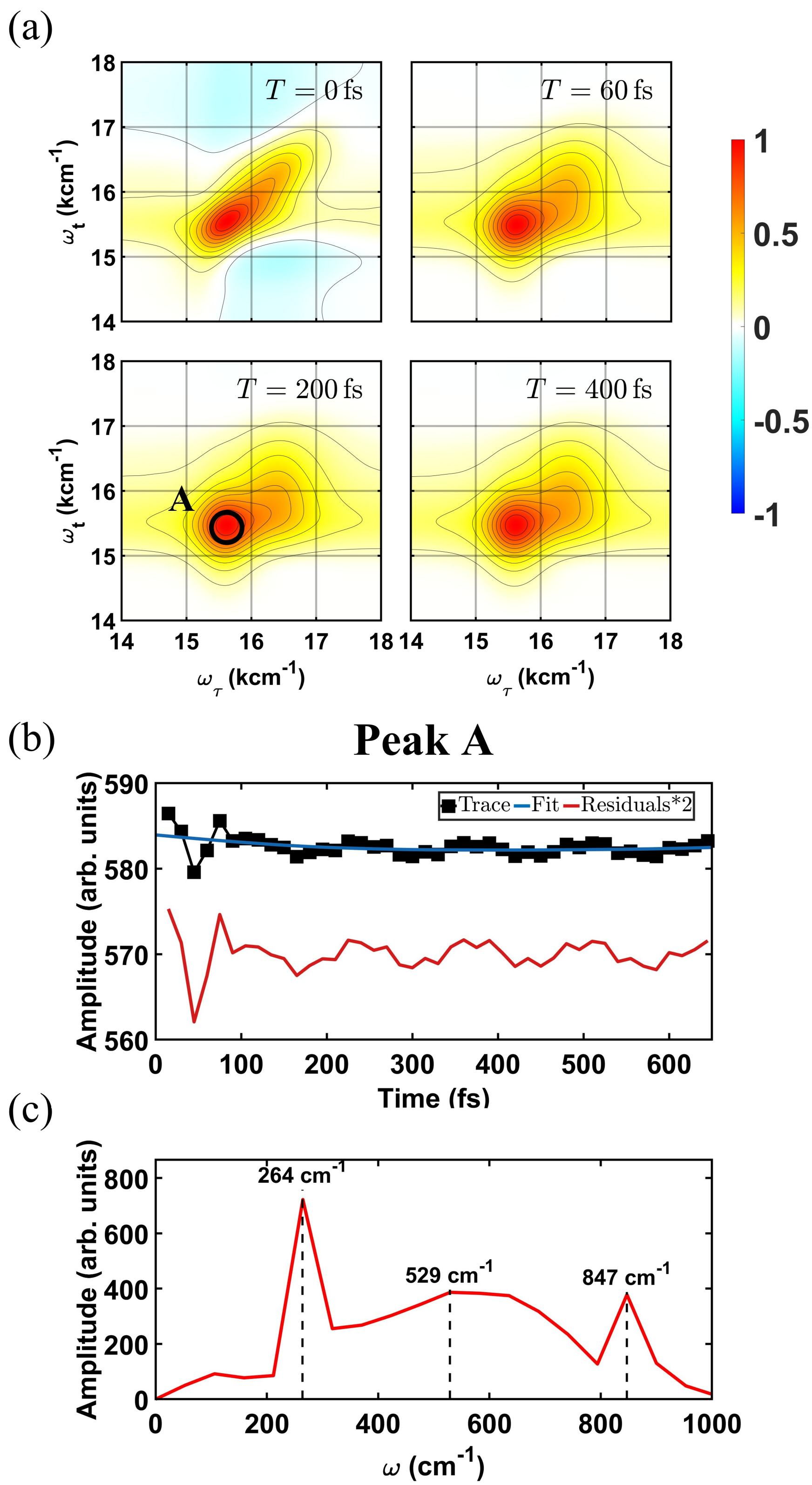

FIG. S22. Theoretical 2DES of the APC monomer. (a) Calculated real part of the APC-m 2DES with different waiting times (T = 0, 60, 200 and 400 fs). (b) Simulated time trace, multi-exponential fit, and residuals at peak A. (c) Mode spectrum obtained from the Fourier analysis of the residuals at peak A.

S18. PARAMETER-SPACE ANALYSIS OF ELECTRONIC COHERENCE LIFETIME

To place APC in a broader excitonic context, we extended the model calculations beyond the refined APC parameter set and systematically evaluated the electronic coherence lifetime over a wider parameter space defined by the site energy gap ΔE and the electronic coupling strength J. This analysis was performed to assess how the electronic coherence lifetime depends on the excitonic parameters themselves and to compare APC with other representative photosynthetic complexes within a unified parameter-space framework. Figure S23 summarizes the calculated electronic coherence lifetime as a function of ΔE and J. The parameter regions corresponding to representative photosynthetic complexes, including FMO, LHCII, and APC, are indicated for comparison. The dashed lines mark the boundary between long-lived coherence (> 100 fs) and short-lived coherence (< 100 fs). Although these systems occupy markedly different excitonic regimes, the calculations show that the electronic coherence remains in the short-lived regime throughout the physically relevant parameter range. No pronounced enhancement or protection of the electronic coherence lifetime is observed within the parameter regions relevant to the complexes considered here. This result generalizes the APC-specific conclusion obtained in the main text and in Sec. XV. While variations in excitonic parameters can modify the spectral appearance of oscillatory signals and can lead to mixed vibronic features, they do not produce long-lived electronic coherence in the present model. APC is therefore not an isolated exception, but part of a broader parameter-space trend in which photosynthetic complexes with substantially different energy gaps and coupling strengths still exhibit similarly rapid electronic decoherence. The parameter-space analysis further supports the central conclusion of this work: long-lived oscillatory signals observed in APC should not be interpreted as evidence for sustained electronic coherence. Instead, the electronic phase memory remains much shorter than the energy-transfer timescale across the relevant parameter regimes. This reinforces the view that the dominant long-lived beatings in APC originate primarily from vibrational dynamics rather than from functionally relevant long-lived electronic superpositions.

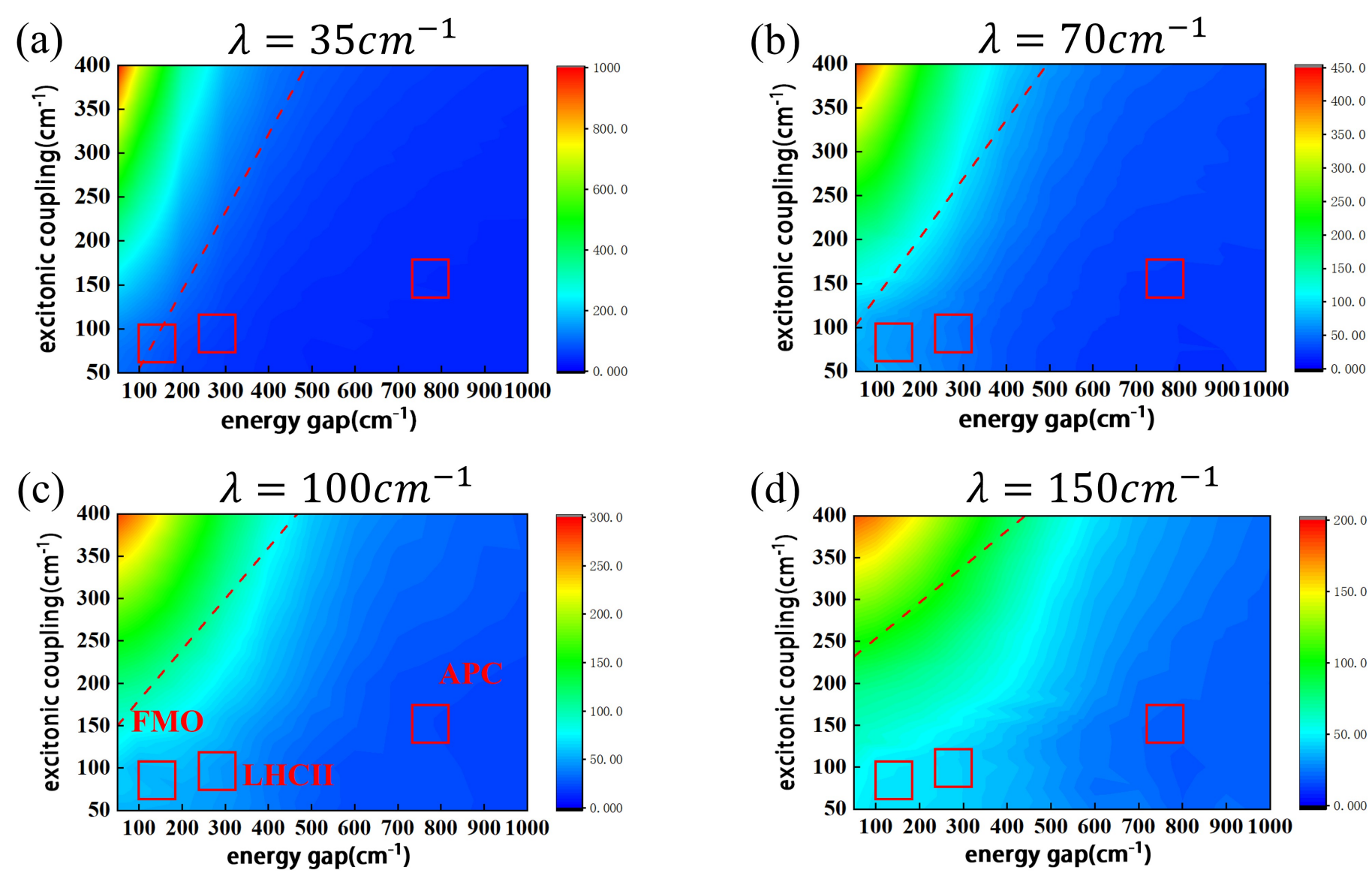

FIG. S23. Parameter-space mapping of electronic coherence lifetime in photosynthetic complexes. Calculated electronic coherence lifetimes are plotted as a function of site energy gap ΔE and electronic coupling strength J. The marked regions indicate the representative parameter regimes of FMO, LHCII, and APC complexes. The dashed lines mark the boundary between long-lived coherence (> 100 fs) and short-lived coherence (< 100 fs). Panels (a)–(d) correspond to four representative calculation conditions with different reorganization energies in the Drude spectral density: (a) $\lambda = 35\ \mathrm{cm}^{-1}$; (b) $\lambda = 70\ \mathrm{cm}^{-1}$; (c) $\lambda = 100\ \mathrm{cm}^{-1}$; (d) $\lambda = 150\ \mathrm{cm}^{-1}$.

S19. EFFECT OF ADDITIONAL VIBRATIONAL-BATH COUPLING ON ENERGY-TRANSFER DYNAMICS

To further examine whether long-lived vibrational coherence itself plays a dominant role in controlling the energy-transfer rate, we carried out an additional control calculation based on the same refined APC model used throughout

this work. The model retains the two intramolecular vibrational modes at 250 and 800 cm^{-1} and the same standard bath parameters as in the main calculations. In addition, we introduced two auxiliary baths that couple directly to the vibrational subspace. In the matrix representation, these additional couplings act on two vibrational-coordinate operators, denoted by

$$\hat{Q}_{t1} = I_{\text{elec}} \otimes I_{t2} \otimes q_{t1}, \qquad \hat{Q}_{t2} = I_{\text{elec}} \otimes q_{t2} \otimes I_{t1},$$

where q_{t1} and q_{t2} are the coordinate operators of the two vibrational subspaces, and I_{elec}, I_{t1}, and I_{t2} are identity operators in the corresponding electronic and vibrational spaces. In this way, the extra environmental dissipation is applied directly to the vibrational degrees of freedom rather than to the electronic populations. For these two auxiliary vibrational baths, we set the reorganization energies to $\lambda = 30$, 50, and 100 cm^{-1}, while keeping all other Hamiltonian and bath parameters unchanged. The resulting population-transfer dynamics are shown in Fig. S24. Using the same fitting procedure as in Fig. S16, we obtained characteristic transfer times of $\tau = 160.622$, 164.002, and 170.258 fs for $\lambda = 30$, 50, and 100 cm^{-1}, respectively. These results show that, even when the vibrational-bath coupling strength is increased over this range, the energy-transfer time changes only weakly. Within the present APC model, this additional control calculation therefore does not support long-lived vibrational coherence as the dominant factor governing the energy-transfer timescale. Instead, the long-lived vibrational beatings mainly reflect nuclear dynamics that leave a clear spectroscopic signature, while the overall population-transfer rate remains nearly unchanged upon varying the vibrational-bath coupling.

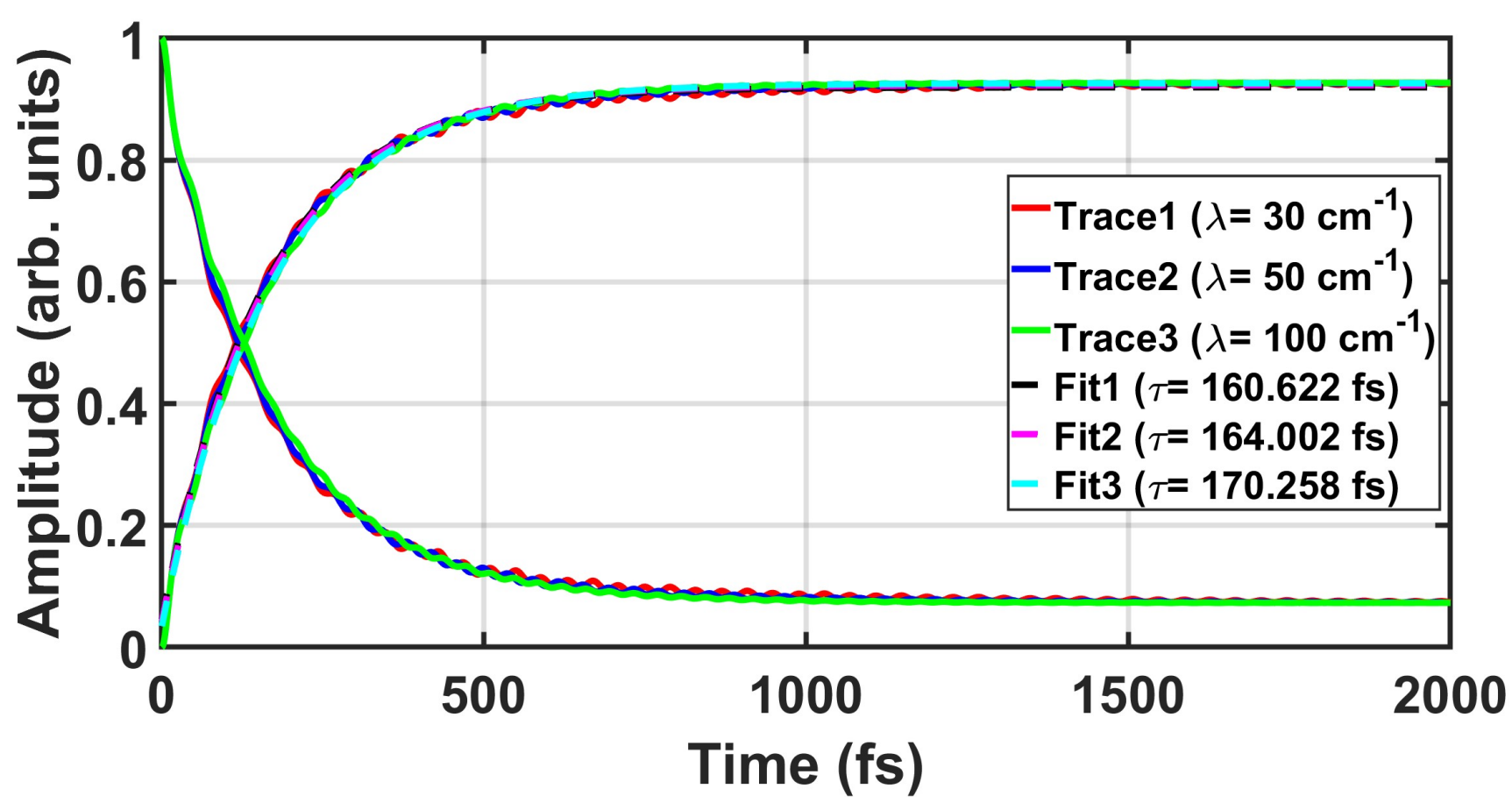

FIG. S24. Population-transfer dynamics calculated with two additional baths coupled to the vibrational-coordinate subspaces of the refined APC model. The auxiliary vibrational baths are characterized by reorganization energies of $\lambda = 30$, 50, and 100 cm^{-1}. Dashed curves show fits obtained using the same procedure as in Fig. S16, yielding transfer times of $\tau = 160.622$ fs, 164.002 fs, and 170.258 fs, respectively. The weak variation of the fitted transfer time indicates that increasing the environmental coupling of the vibrational coordinates produces only a marginal change in the energy transfer rate.

S20. CALCULATED OPTICAL DEPHASING DYNAMICS AT 80 K AND 300 K

To further validate the interpretation of the anti-diagonal linewidth analysis, we calculated the optical dephasing dynamics between the ground and excited states using the refined vibronic dimer Hamiltonian within the HEOM framework. The calculated optical dephasing profiles at 80 K and 300 K are shown in Fig. S25. The extracted full widths at half maximum are 224.80 cm^{-1} and 283.60 cm^{-1}, corresponding to homogeneous optical dephasing times of approximately 45 fs and 35 fs, respectively. These values are consistent with the experimentally determined anti-diagonal linewidth analysis and support the use of the refined model for describing the optical dephasing dynamics of APC. Fully converged HEOM calculations at 10 K require substantially larger hierarchy depths because of the longer bath correlation time at cryogenic temperature; therefore, only the 80 K and 300 K calculations are presented here.

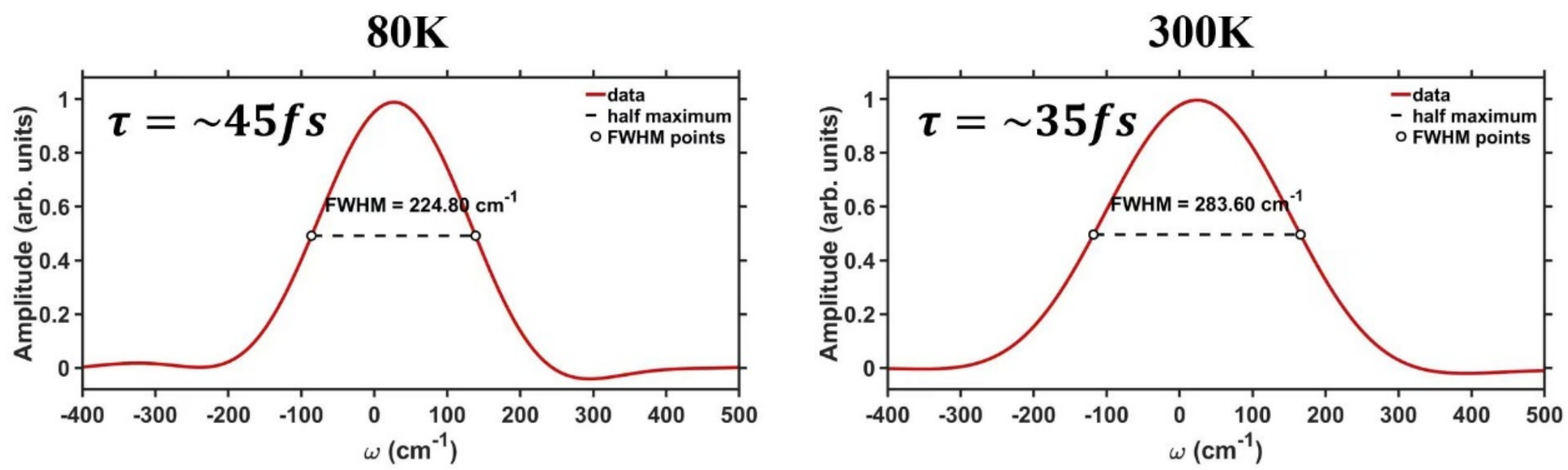

FIG. S25. Calculated optical dephasing of the APC excitonic transition obtained using the refined vibronic dimer model within the HEOM framework at 80 K and 300 K. The red curves represent the calculated optical coherence decay, the dashed lines indicate the half-maximum level, and the open circles mark the FWHM points. The extracted FWHM values of 224.80 cm^{-1} and 283.60 cm^{-1} correspond to homogeneous optical dephasing times of approximately 45 fs and 35 fs, respectively.

REFERENCES

1. P. Jordan, *Die Physik und das Geheimnis des organischen Lebens* (F. Vieweg, 1941).

2. E. Schrodinger, *What is Life?: With Mind and Matter and Autobiographical Sketches* (Cambridge Univ. Press, 2014).

3. R. E. Blankenship, *Molecular Mechanisms of Photosynthesis* (Wiley, 2021).

4. N. Nelson, W. Junge, Structure and energy transfer in photosystems of oxygenic photosynthesis. *Annu. Rev. Biochem.* **84**, 659–683 (2015).

5. A. Stirbet, D. Lazár, Y. Guo, G. Govindjee, Photosynthesis: Basics, history and modelling. *Ann. Bot.* **126**, 511–537 (2020).

6. A. Marais, B. Adams, A. K. Ringsmuth, M. Ferretti, J. M. Gruber, R. Hendrikx, M. Schuld, S. L. Smith, I. Sinayskiy, T. P. J. Krüger, F. Petruccione, R. van Grondelle, The future of quantum biology. *J. R. Soc. Interface* **15**, 20180640 (2018).

7. N. Lambert, Y.-N. Chen, Y.-C. Cheng, C.-M. Li, G.-Y. Chen, F. Nori, Quantum biology. *Nat. Phys.* **9**, 10–18 (2013).

8. J. Cao, R. J. Cogdell, D. F. Coker, H.-G. Duan, J. Hauer, U. Kleinekathöfer, T. L. C. Jansen, T. Mančal, R. J. D. Miller, J. P. Ogilvie, V. I. Prokhorenko, T. Renger, H.-S. Tan, R. Tempelaar, M. Thorwart, E. Thyrhaug, S. Westenhoff, D. Zigmantas, Quantum biology revisited. *Sci. Adv.* **6**, eaaz4888 (2020).

9. A. O. Caldeira, A. J. Leggett, Influence of damping on quantum interference: An exactly soluble model. *Phys. Rev. A* **31**, 1059–1066 (1985).

10. P. C. W. Davies, Does quantum mechanics play a non-trivial role in life? *Biosystems* **78**, 69–79 (2004).

11. W. G. Unruh, W. H. Zurek, Reduction of a wave packet in quantum Brownian motion. *Phys. Rev. D* **40**, 1071–1094 (1989).

12. U. Weiss, *Quantum Dissipative Systems* (World Scientific, 1999).

13. H. P. Breuer, F. Petruccione, *The Theory of Open Quantum Systems* (Oxford Univ. Press, 2003).

14. F. D. Fuller, J. P. Ogilvie, Experimental implementations of two-dimensional Fourier transform electronic spectroscopy. *Annu. Rev. Phys. Chem.* **66**, 667–690 (2015).

15. E. Fresch, F. V. A. Camargo, Q. Shen, C. C. Bellora, T. Pullerits, G. S. Engel, G. Cerullo, E. Collini, Two-dimensional electronic spectroscopy. *Nat. Rev. Methods Primers* **3**, 84 (2023).

16. M. Cho, Coherent two-dimensional optical spectroscopy. *Chem. Rev.* **108**, 1331–1418 (2008).

17. G. S. Engel, T. R. Calhoun, E. L. Read, T.-K. Ahn, T. Mančal, Y.-C. Cheng, R. E. Blankenship, G. R. Fleming, Evidence for wavelike energy transfer through quantum coherence in photosynthetic systems. *Nature* **446**, 782–786 (2007).

18. E. Collini, C. Y. Wong, K. E. Wilk, P. M. G. Curmi, P. Brumer, G. D. Scholes, Coherently wired light-harvesting in photosynthetic marine algae at ambient temperature. *Nature* **463**, 644–647 (2010).

19. V. Tiwari, W. K. Peters, D. M. Jonas, Electronic resonance with anticorrelated pigment vibrations drives photosynthetic energy transfer outside the adiabatic framework. *Proc. Natl. Acad. Sci. U.S.A.* **110**, 1203–1208 (2013).

20. J. Piilo, S. Maniscalco, K. Härkönen, K.-A. Suominen, Non-Markovian quantum jumps. *Phys. Rev. Lett.* **100**, 180402 (2008).

21. H. Lee, Y.-C. Cheng, G. R. Fleming, Coherence dynamics in photosynthesis: Protein protection of excitonic coherence. *Science* **316**, 1462–1465 (2007).

22. D. S. Hoffmann, P. M. Dohmen, M. Sokolov, U. Kleinekathöfer, M. Elstner, Exciton transfer simulations in a light-harvesting 2 complex reveal the transient delocalization mechanism. *J. Phys. Chem. B* **129**, 3345–3365 (2025).

23. T. Renger, M. E.-A. Madjet, M. Schmidt am Busch, J. Adolphs, F. Müh, Structure-based modeling of energy transfer in photosynthesis. *Photosynth. Res.* **116**, 367–388 (2013).

24. C. Curutchet, B. Mennucci, Quantum chemical studies of light harvesting. *Chem. Rev.* **117**, 294–343 (2017).

25. A. Sirohiwal, D. A. Pantazis, Reaction center excitation in photosystem II: From multiscale modeling to functional principles. *Acc. Chem. Res.* **56**, 2921–2932 (2023).

26. S. Maity, U. Kleinekathöfer, Recent progress in atomistic modeling of light-harvesting complexes: A mini review. *Photosynth. Res.* **156**, 147–162 (2023).

27. H.-G. Duan, V. I. Prokhorenko, R. J. Cogdell, K. Ashraf, A. L. Stevens, M. Thorwart, R. J. D. Miller, Nature does not rely on long-lived electronic quantum coherence for photosynthetic energy transfer. *Proc. Natl. Acad. Sci. U.S.A.* **114**, 8493–8498 (2017).

28. D. Paleček, P. Edlund, S. Westenhoff, D. Zigmantas, Quantum coherence as a witness of vibronically hot energy transfer in bacterial reaction center. *Sci. Adv.* **3**, e1603141 (2017).

29. E. Thyrhaug, R. Tempelaar, M. J. P. Alcocer, K. Žídek, D. Bína, J. Knoester, T. L. C. Jansen, D. Zigmantas, Identification and characterization of diverse coherences in the Fenna-Matthews-Olson complex. *Nat. Chem.* **10**, 780–786 (2018).

30. H.-G. Duan, A. Jha, L. Chen, V. Tiwari, R. J. Cogdell, K. Ashraf, V. I. Prokhorenko, M. Thorwart, R. J. D. Miller, Quantum coherent energy transport in the Fenna-Matthews-Olson complex at low temperature. *Proc. Natl. Acad. Sci. U.S.A.* **119**, e2212630119 (2022).

31. A. Jha, P.-P. Zhang, V. Tiwari, L. Chen, M. Thorwart, R. J. D. Miller, H.-G. Duan, Unraveling quantum coherences mediating primary charge transfer processes in photosystem II reaction center. *Sci. Adv.* **10**, eadk1312 (2024).

32. A. Halpin, P. J. M. Johnson, R. Tempelaar, R. S. Murphy, J. Knoester, T. L. C. Jansen, R. J. D. Miller, Two-dimensional spectroscopy of a molecular dimer unveils the effects of vibronic coupling on exciton coherences. *Nat. Chem.* **6**, 196–201 (2014).

33. J. E. Runeson, D. E. Manolopoulos, Nuclear quantum effects slow down the energy transfer in biological light-harvesting complexes. *Sci. Adv.* **11**, eadw4798 (2025).

34. E. Zerah Harush, Y. Dubi, Do photosynthetic complexes use quantum coherence to increase their efficiency? Probably not. *Sci. Adv.* **7**, eabc4631 (2021).

35. V. Butkus, J. Alster, E. Bašinskaite, R. Augulis, P. Neuhaus, L. Valkunas, H. L. Anderson, D. Abramavicius, D. Zigmantas, Discrimination of diverse coherences allows identification of electronic transitions of a molecular nanoring. *J. Phys. Chem. Lett.* **8**, 2344–2349 (2017).

36. J. Seibt, T. Pullerits, Beating signals in 2D spectroscopy: Electronic or nuclear coherences? Application to a quantum dot model system. *J. Phys. Chem. C* **117**, 18728–18737 (2013).

37. D. Egorova, Self-analysis of coherent oscillations in time-resolved optical signals. *J. Phys. Chem. A* **118**, 10259–10267 (2014).

38. K. Brejc, R. Ficner, R. Huber, S. Steinbacher, Isolation, crystallization, crystal structure analysis and refinement of allophycocyanin from the cyanobacterium Spirulina platensis at 2.3 Å resolution. *J. Mol. Biol.* **249**, 424–440 (1995).

39. M. Watanabe, M. Ikeuchi, Phycobilisome: Architecture of a light-harvesting supercomplex. *Photosynth. Res.* **116**, 265–276 (2013).

40. B. A. Diner, F. Rappaport, Structure, dynamics, and energetics of the primary photochemistry of photosystem II of oxygenic photosynthesis. *Annu. Rev. Plant Biol.* **53**, 551–580 (2002).

41. A. N. Glazer, Light guides: Directional energy transfer in a photosynthetic antenna. *J. Biol. Chem.* **264**, 1–4 (1989).

42. J. M. Womick, A. M. Moran, Vibronic enhancement of exciton sizes and energy transport in photosynthetic complexes. *J. Phys. Chem. B* **115**, 1347–1356 (2011).

43. J. M. Womick, A. M. Moran, Exciton coherence and energy transport in the light-harvesting dimers of allophycocyanin. *J. Phys. Chem. B* **113**, 15747–15759 (2009).

44. J. M. Womick, A. M. Moran, Nature of excited states and relaxation mechanisms in C-phycocyanin. *J. Phys. Chem. B* **113**, 15771–15782 (2009).

45. J. M. Womick, B. A. West, N. F. Scherer, A. M. Moran, Vibronic effects in the spectroscopy and dynamics of C-phycocyanin. *J. Phys. B. At. Mol. Opt. Phys.* **45**, 154016 (2012).

46. B. J. Homoelle, M. D. Edington, W. M. Diffey, W. F. Beck, Stimulated photon-echo and transient-grating studies of protein-matrix solvation dynamics and interexciton-state radiationless decay in α-phycocyanin and allophycocyanin. *J. Phys. Chem. B* **102**, 3044–3052 (1998).

47. R. Zhu, W. Li, Z. Zhen, J. Zou, G. Liao, J. Wang, Z. Wang, H. Chen, S. Qin, Y. Weng, Quantum phase synchronization via exciton-vibrational energy dissipation sustains long-lived coherence in photosynthetic antennas. *Nat. Commun.* **15**, 3171 (2024).

48. S. Long, M. Zhou, K. Tang, X.-L. Zeng, Y. Niu, Q. Guo, K.-H. Zhao, A. Xia, Single-molecule spectroscopy and femtosecond transient absorption studies on the excitation energy transfer process in ApcE(1-240) dimers. *Phys. Chem. Chem. Phys.* **17**, 13387–13396 (2015).

49. R. MacColl, Allophycocyanin and energy transfer. *Biochim. Biophys. Acta Bioenerg.* **1657**, 73–81 (2004).

50. D. M. Jonas, Vibrational and nonadiabatic coherence in 2D electronic spectroscopy, the Jahn-Teller effect, and energy transfer. *Annu. Rev. Phys. Chem.* **69**, 327–352 (2018).

51. A. Jha, F. Zheng, Z. Liu, S. Mukamel, M. Thorwart, R. J. D. Miller, H.-G. Duan, Quantum coherent dynamics in photosynthetic protein complexes. *Chem. Soc. Rev.* **55**, 1089–1130 (2026).

52. X. Zhang, J. Zhou, F. Zheng, B. Yang, A. Jha, H.-G. Duan, Ultrafast exciton-phonon coupling and energy transfer dynamics in quasi-2D layered Ruddlesden-Popper perovskites. *Commun. Phys.* **8**, 71 (2025).

53. J. Yang, P. Zhang, Z. Liu, M. Penny, S. Mosca, J. Zhou, V. Tiwari, B. G. Davis, F. Zheng, H.-G. Duan, A. Jha, Mapping vibronic dynamics of ultrafast intersystem crossing in an earth-abundant ligand-field excited complex. *J. Am. Chem. Soc.* **148**, 1977–1988 (2026).

54. J. D. Hybl, A. Albrecht Ferro, D. M. Jonas, Two-dimensional Fourier transform electronic spectroscopy. *J. Chem. Phys.* **115**, 6606–6622 (2001).

55. V. I. Prokhorenko, Global analysis of multi-dimensional experimental data. *Eur. Photochem. Assoc. Newsletter*, 21–23 (2012).

56. A. Cohen, J. Kovačevič, Wavelets: The mathematical background. *Proc. IEEE* **84**, 514–522 (1996).

57. J. D. Harrop, S. N. Taraskin, S. R. Elliott, Instantaneous frequency and amplitude identification using wavelets: Application to glass structure. *Phys. Rev. E* **66**, 026703 (2002).

58. J. C. van den Berg, *Wavelets in Physics* (Cambridge Univ. Press, 2010).
